# Supplementary material for: Epidemiology, comorbidities, treatments and outcomes of autoimmune liver diseases: A French nationwide study
Source: JHEP Rep. 2025 Aug 11;7(11):101546. doi: 10.1016/j.jhepr.2025.101546 (PMC12519275; doi:10.1016/j.jhepr.2025.101546)
Supplement: Multimedia component 1 [file mmc1.pdf]

# Epidemiology, comorbidities, treatments and outcomes of autoimmune liver diseases: A French nationwide study

Christophe Corpechot, Pierre Hornus, Mallory Cals, Pierre Rinder, Théo Marcille, Amina Malek, Karima Ben Belkacem, Farid Gaouar, Yasmina Chabane, Pierre Corret, Paola Squarzoni, Pierre Antoine Soret, Sara Lemoinne, Olivier Chazouillères, Angela Leburgue

Table of contents

Fig. S1 ........................................ 2

Fig. S2 ........................................ 3

Fig. S3. ........................................ 4

Fig. S4. ........................................ 5

Fig. S5. ........................................ 6

Fig. S6 ........................................ 7

Fig. S7 ........................................ 8

Table S1. ........................................ 9

Table S2 ........................................ 10

Table S3. ........................................ 11

Table S4 ........................................ 12

Table S5 ........................................ 13

List of codes used for analysis. ........................................ 14

**Fig. S1. Flow chart of the study.**

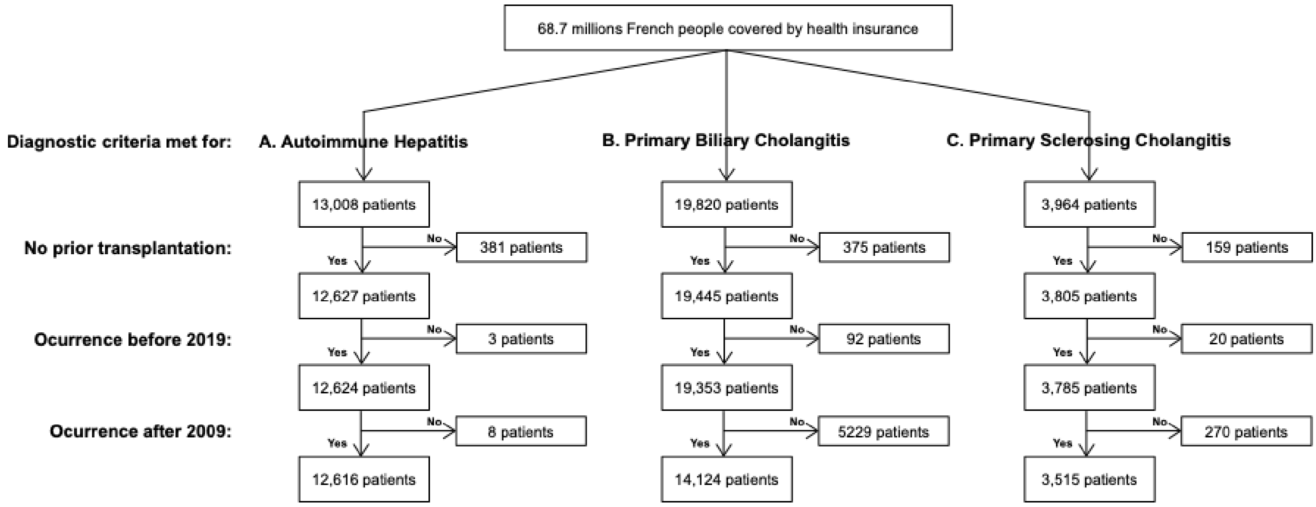

**Fig. S2. Moran scatterplots of French departments for the prevalence of the three AILDs.**

**A. Autoimmune Hepatitis:**

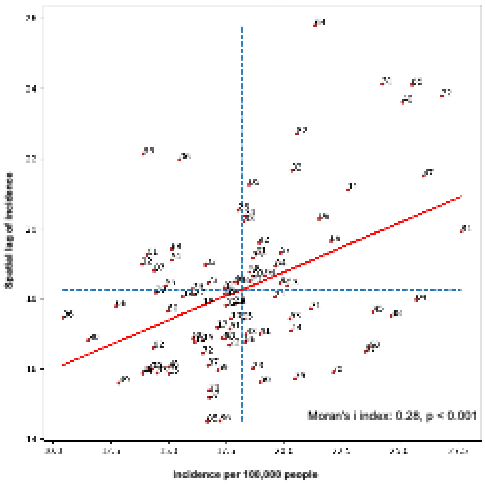


**B. Primary Biliary Cholangitis:**

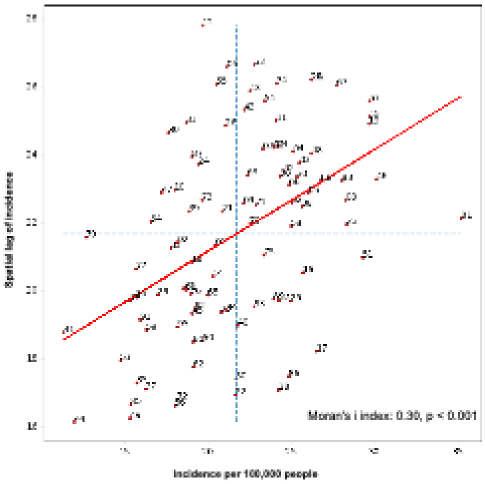


**C. Primary Sclerosing Cholangitis:**

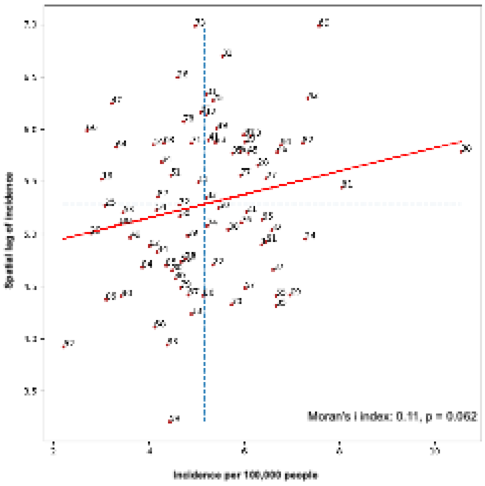

**Fig. S3. Correlation between departmental prevalence and local density of hepato-gastroenterologists.**

**A. Autoimmune Hepatitis:**

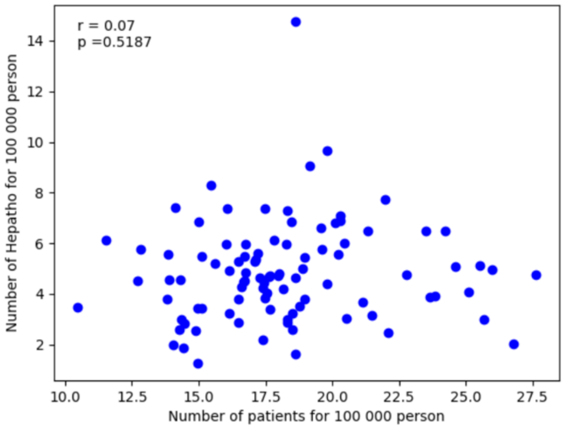


**B. Primary Biliary Cholangitis:**

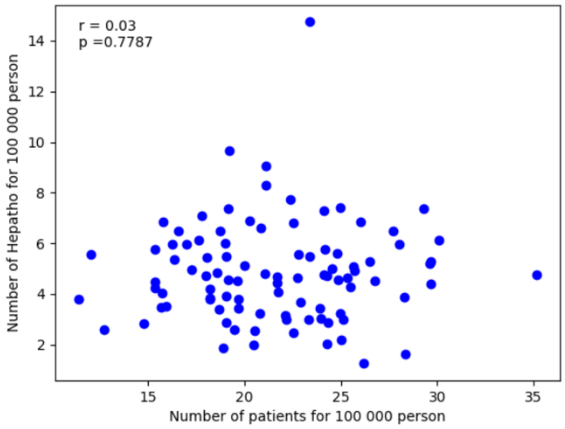


**C. Primary Sclerosing Cholangitis:**

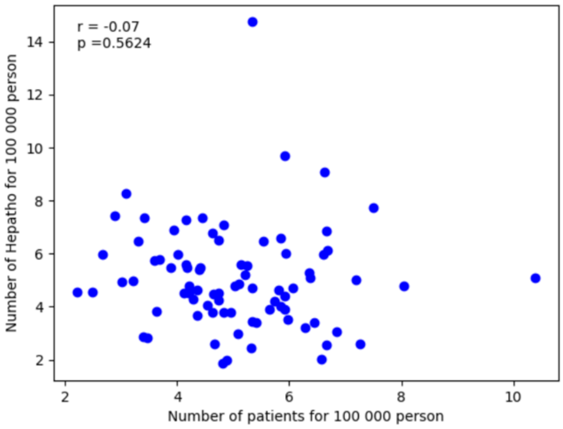

**Fig. S4. Distribution of the number of consultations in the year following inclusion.**

**A. Autoimmune Hepatitis:**

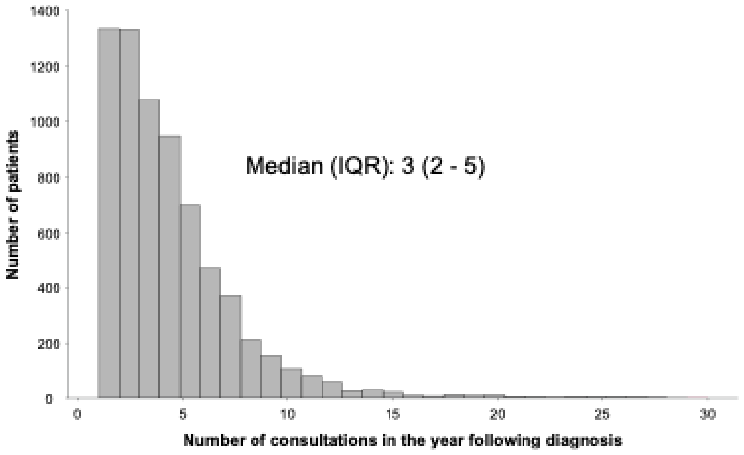


**B. Primary Biliary Cholangitis:**

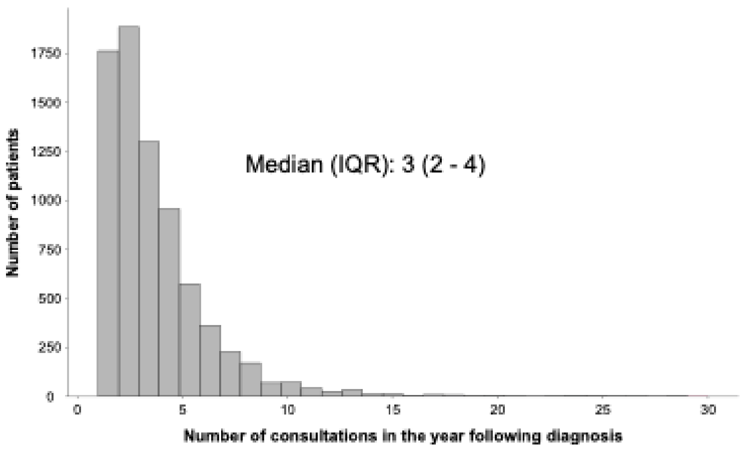


**C. Primary Sclerosing Cholangitis:**

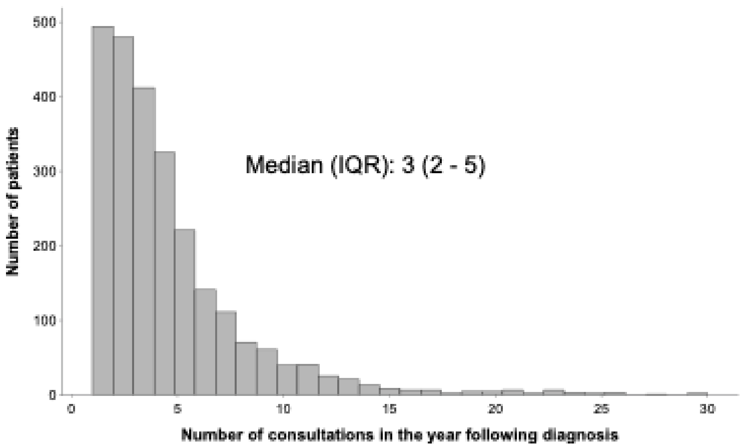

**Fig. S5. Distribution of the number of hospital admissions in the year following inclusion.**

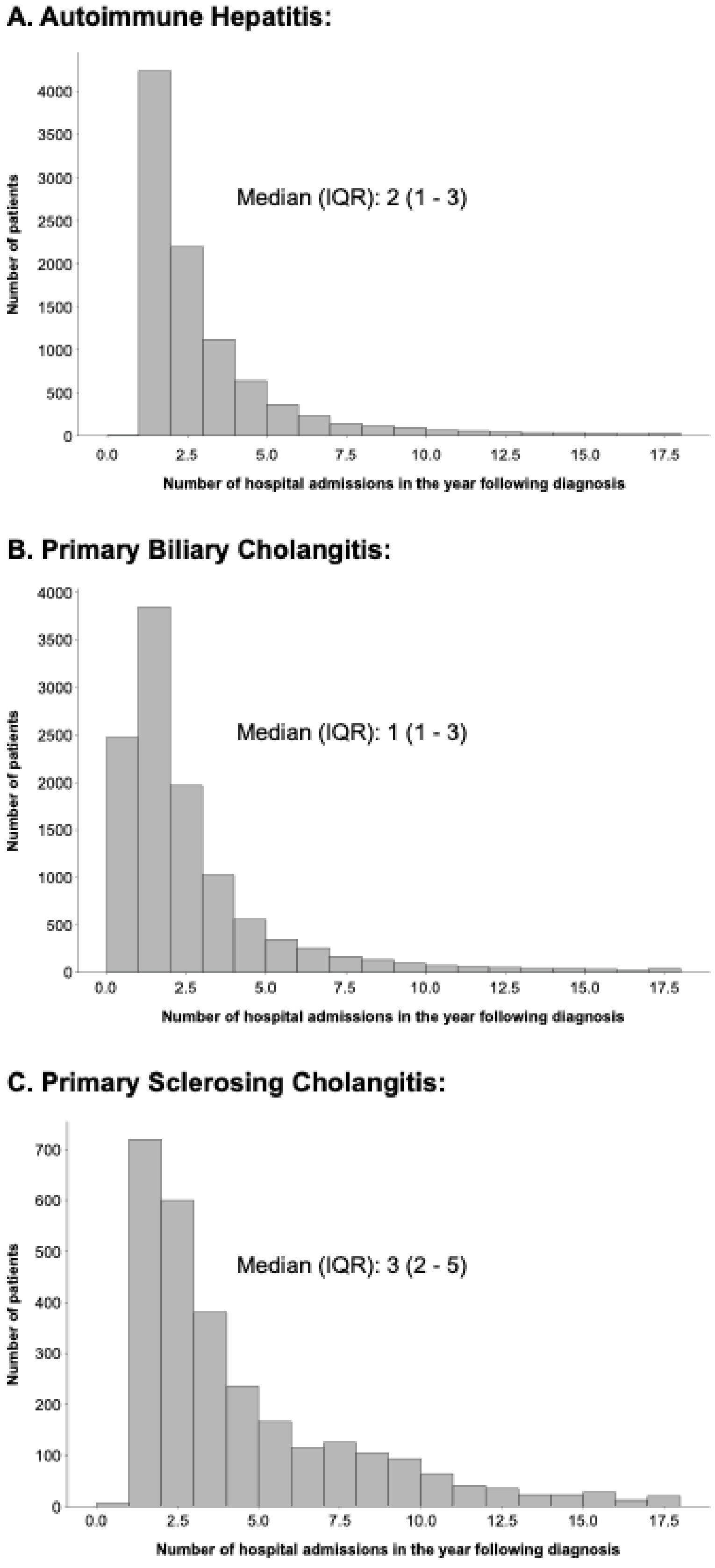

**Fig. S6. Distribution of the number of inpatient days in the year following inclusion.**

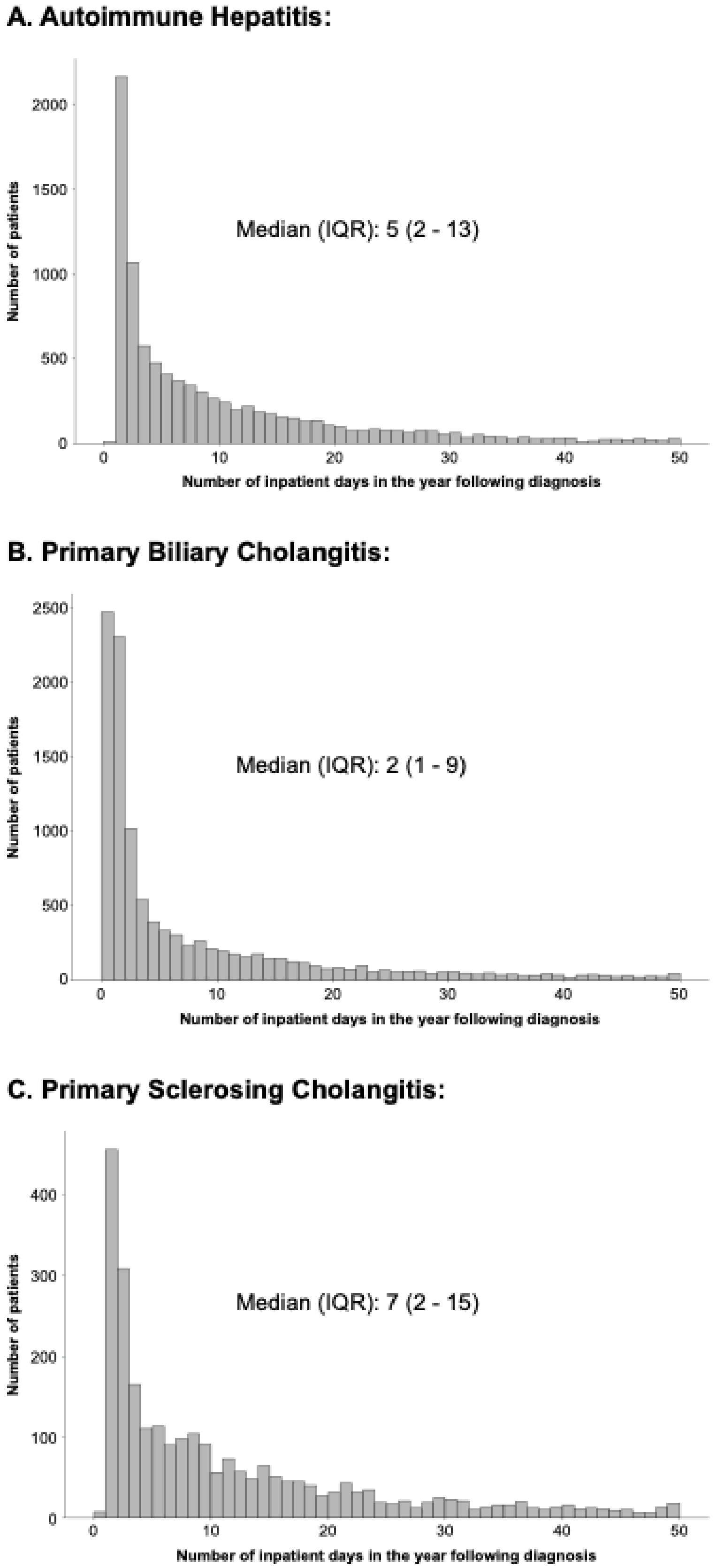

**Fig. S7. Alluvial diagram of drugs dispensed in the year following inclusion.**

**A. Autoimmune Hepatitis:**

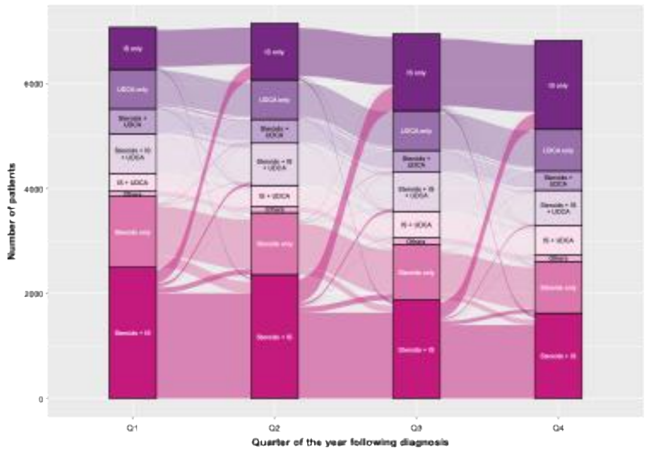


**B. Primary Biliary Cholangitis:**

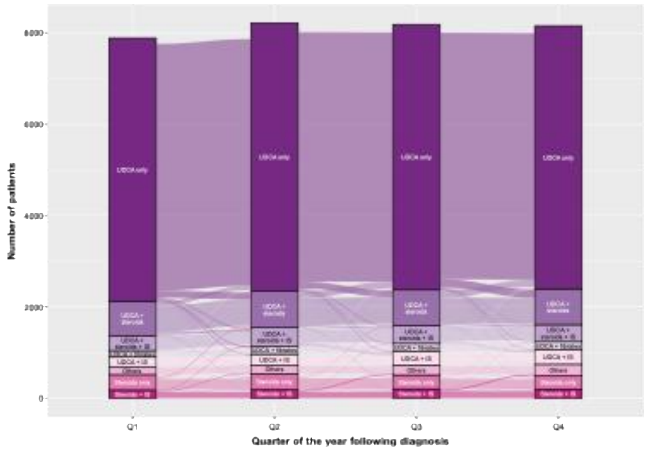


**C. Primary Sclerosing Cholangitis:**

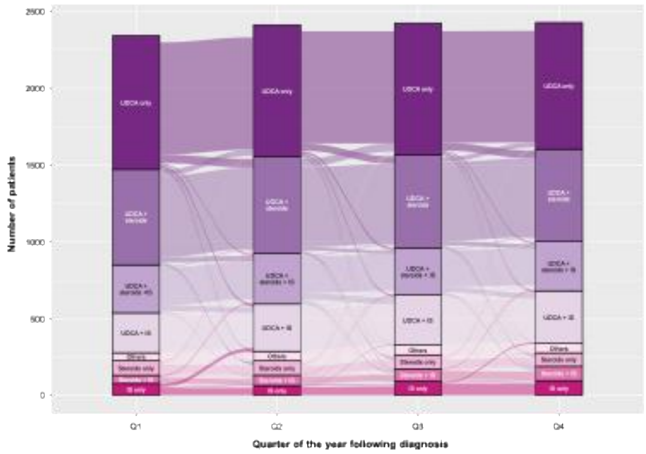

**Table S1. Overall and disease-specific prevalence (percentage) of extrahepatic autoimmune conditions at the time of AILD diagnosis.**

| Condition | Overall | AIH | PBC | PSC |
|---|---|---|---|---|
| Autoimmune thyroiditis | 2.93 | 3.50 | 2.70 | 1.80 |
| Type 1 diabetes | 2.52 | 2.29 | 2.83 | 2.09 |
| Sjögren’s syndrome | 1.21 | 1.39 | 1.20 | 0.55 |
| Systemic scleroderma | 1.16 | 0.69 | 1.75 | 0.46 |
| Rheumatoid arthritis | 1.04 | 1.20 | 0.97 | 0.78 |
| Systemic lupus erythematosus | 0.73 | 1.20 | 0.42 | 0.32 |
| Raynaud’s syndrome | 0.69 | 0.55 | 0.91 | 0.29 |
| Ankylosing spondylitis | 0.47 | 0.62 | 0.27 | 0.73 |
| Psoriasis | 0.44 | 0.55 | 0.35 | 0.41 |
| Multiple sclerosis | 0.34 | 0.48 | 0.24 | 0.20 |
| Imm. thrombocytopenic purpura | 0.26 | 0.32 | 0.22 | 0.17 |
| Pernicious anemia | 0.22 | 0.23 | 0.23 | 0.12 |
| Celiac disease | 0.21 | 0.29 | 0.13 | 0.23 |
| Dermatomyositis / Polymyositis | 0.18 | 0.25 | 0.14 | 0.06 |

Autoimmune diseases are ranked in descending order of overall prevalence. Those with an overall prevalence of less than 0.1% were excluded.

**Table S2. The 10 most frequently reported active cancer codes in the year prior to inclusion.**

| | Designation | Occurrences |
|---|---|---|
| **AIH** | | |
| C780 | Secondary malignant lung tumor | 76 |
| C787 | Secondary malignant tumor of the liver and intrahepatic bile ducts | 70 |
| C349 | Malignant tumor of bronchus or lung, unspecified | 62 |
| C795 | Secondary malignant tumor of bone and bone marrow | 52 |
| C439 | Melanoma of the skin | 45 |
| C792 | Secondary malignant skin tumor | 44 |
| C509 | Malignant breast tumor, unspecified | 43 |
| C793 | Secondary malignant tumor of the brain and cerebral meninges | 40 |
| C435 | Malignant melanoma of the trunk | 38 |
| C786 | Secondary malignant tumor of the retroperitoneum and peritoneum | 38 |
| **PBC** | | |
| C509 | Malignant breast tumor, unspecified | 48 |
| C787 | Secondary malignant tumor of the liver and intrahepatic bile ducts | 37 |
| C61 | Malignant prostate tumor | 29 |
| C443 | Malignant tumor of the skin of the face, other and unspecified parts | 23 |
| C349 | Malignant tumor of bronchus or lung, unspecified | 22 |
| C187 | Malignant tumor of the sigmoid colon | 22 |
| C780 | Secondary malignant lung tumor | 21 |
| C795 | Secondary malignant tumor of bone and bone marrow | 21 |
| C50 | Malignant breast tumor | 21 |
| C786 | Secondary malignant tumor of the retroperitoneum and peritoneum | 20 |
| **PSC** | | |
| C189 | Malignant tumor of the colon, unspecified | 13 |
| C249 | Malignant tumor of the bile ducts, unspecified | 11 |
| C787 | Secondary malignant tumor of the liver and intrahepatic bile ducts | 9 |
| C20 | Malignant tumor of the rectum | 9 |
| C187 | Malignant tumor of the sigmoid colon | 7 |
| C779 | Secondary, unspecified malignant tumor of a lymph node | 7 |
| C180 | Malignant tumor of the cecum | 6 |
| C509 | Malignant breast tumor, unspecified | 6 |
| C786 | Secondary malignant tumor of the retroperitoneum and peritoneum | 6 |

**Table S3. Number of medicines dispensed and users in the year following inclusion (patients aged over 18 years with one year of data available after inclusion).**

| | No. of deliveries | No. of users | Percent. of users |
|---|---|---|---|
| **AIH (n=9,599)** | | | |
| Ursodeoxycholic acid | 26,063 | 2,996 | 31.2% |
| Obeticholic acid | 78 | 11 | 0.1% |
| Bezafibrate/fenofibrate | 1,028 | 186 | 1.9% |
| Corticosteroids | 47,399 | 5,729 | 59.7% |
| Prednisone | 29,972 | 3,533 | 36.8% |
| Prednisolone | 13,119 | 2,729 | 28.4% |
| Budesonide | 7,910 | 1,565 | 16.3% |
| Immunosuppressives | 43,820 | 5,853 | 59.9% |
| Azathioprine | 31,571 | 4,940 | 51.5% |
| Mycophenolate mofetil | 5,446 | 743 | 7.7% |
| Tacrolimus | 2,312 | 218 | 2.3% |
| Ciclosporin | 1,497 | 149 | 1.6% |
| **PBC (n=10,905)** | | | |
| Ursodeoxycholic acid | 79,809 | 7,938 | 72.8% |
| Obeticholic acid | 132 | 31 | 0.3% |
| Bezafibrate/fenofibrate | 2,085 | 359 | 3.3% |
| Corticosteroids | 17,607 | 3,680 | 33.8% |
| Prednisone | 10,555 | 1,450 | 13.3% |
| Prednisolone | 4,648 | 1,987 | 18.2% |
| Budesonide | 1,791 | 363 | 3.3% |
| Immunosuppressives | 10,971 | 1,319 | 11.7% |
| Azathioprine | 4,357 | 725 | 6.7% |
| Mycophenolate mofetil | 1,675 | 240 | 2.2% |
| Tacrolimus | 1,277 | 110 | 1.0% |
| Ciclosporin | 525 | 51 | 0.5% |
| **PSC (n=2,844)** | | | |
| Ursodeoxycholic acid | 22,457 | 2,534 | 89.1% |
| Obeticholic acid | 61 | ≤ 10 | NA |
| Bezafibrate/fenofibrate | 530 | 88 | 3.1% |
| Corticosteroids | 6,624 | 1,239 | 43.6% |
| Prednisone | 3,520 | 573 | 20.2% |
| Prednisolone | 2,305 | 695 | 24.4% |
| Budesonide | 947 | 203 | 7.1% |
| Immunosuppressives | 9,787 | 1,184 | 35.7% |
| Azathioprine | 3,782 | 577 | 20.3% |
| Mycophenolate mofetil | 939 | 150 | 5.3% |
| Tacrolimus | 1,055 | 112 | 3.9% |
| Ciclosporin | 391 | 35 | 1.2% |

**Table S4. Survival rates and transplant-free survival rates after excluding patients whose follow-up since diagnosis was ≤ 1 year (landmark analysis).**

| | At 5 years | At 10 years |
|---|---|---|
| **AIH** | | |
| Survival rate | 89% (89% – 90%) | 77% (75% – 79%) |
| Transplant-free survival rate | 88% (87% – 89%) | 75% (73% – 76%) |
| **PBC** | | |
| Survival rate | 84% (83% – 85%) | 70% (68% – 72%) |
| Transplant-free survival rate | 82% (82% – 83%) | 68% (66% – 69%) |
| **PSC** | | |
| Survival rate | 90% (88% – 91%) | 78% (75% – 81%) |
| Transplant-free survival rate | 82% (81% – 84%) | 66% (63% – 69%) |

**Table S5. The 10 most frequently reported diagnoses in death certificates (patients whose death certificate included at least one ICD10 code for liver disease)**

| | ICD-10 code | No. of reports | %. of deaths |
|---|---|---|---|
| **AIH (n=593)** | | | |
| Autoimmune hepatitis | K754 | 377 | 30.3% |
| Respiratory arrest | R092 | 206 | 16.6% |
| Hepatic failure | K729 | 170 | 13.7% |
| Unspecified cirrhosis | K746 | 170 | 13.7% |
| Other general symptoms and signs | R688 | 154 | 12.4% |
| Severe sepsis with septic shock | R652 | 110 | 8.9% |
| Sepsis, unspecified organism | A419 | 109 | 8.8% |
| Secondary malignant neoplasm | C799 | 77 | 6.2% |
| Heart failure, unspecified | I509 | 76 | 6.1% |
| Pneumonia, unspecified organism | J189 | 76 | 6.1% |
| **PBC (n=1,061)** | | | |
| Respiratory arrest | R092 | 375 | 17.6% |
| Unspecified cirrhosis | K746 | 369 | 17.3% |
| Primary biliary cirrhosis | K743 | 324 | 15.2% |
| Hepatic failure | K729 | 291 | 13.7% |
| Liver cell carcinoma | C220 | 207 | 9.7% |
| Other general symptoms and signs | R688 | 186 | 8.7% |
| Sepsis, unspecified organism | A419 | 158 | 7.5% |
| Ascites | R18 | 156 | 7.3% |
| Heart failure, unspecified | I509 | 151 | 7.0% |
| Severe sepsis with septic shock | R652 | 133 | 6.2% |
| **PSC (n=125)** | | | |
| Cholangitis | K830 | 63 | 22.9% |
| Intrahepatic bile duct carcinoma | C221 | 44 | 16.0% |
| Hepatic failure | K729 | 41 | 14.9% |
| Unspecified cirrhosis | K746 | 41 | 14.9% |
| Respiratory arrest | R092 | 39 | 14.2% |
| Other general symptoms and signs | R688 | 34 | 12.4% |
| Sepsis, unspecified organism | A419 | 30 | 10.9% |
| Secondary malignant neoplasm | C799 | 27 | 9.8% |
| Ulcerative colitis, unspecified | K519 | 27 | 9.8% |
| Severe sepsis with septic shock | R652 | 24 | 8.7% |

**List of codes used for analysis.**

Codes used to include patients in the PSC cohort

| ICD-10 code | Diagnosis |
|---|---|
| K50 | Crohn's disease |
| K51 | Ulcerative colitis |

| CCAM code | Imaging |
|---|---|
| ZCQN002 | Magnetic resonance imaging of the abdomen or pelvis, without intravenous injection of contrast medium |
| ZCQJ004 | Magnetic resonance imaging of the abdomen or pelvis, with intravenous injection of contrast medium |
| ZCQJ005 | Magnetic resonance imaging of the abdomen and pelvis, with intravenous injection of contrast medium |
| ZCQN001 | Magnetic resonance imaging of the abdomen and pelvis, without intravenous injection of contrast medium |
| ZCQH001 | Computed tomography scan of the abdomen and pelvis, with intravenous injection of contrast medium |
| ZCQH002 | Computed tomography scan of the abdomen or pelvis, with intravenous injection of contrast medium |
| ZCQK004 | Computed tomography scan of the abdomen and pelvis, without intravenous injection of contrast medium |
| ZCQK005 | Computed tomography scan of the abdomen or pelvis, without intravenous injection of contrast medium |
| ZCQM008 | Transcutaneous ultrasound of the abdomen |
| ZCQM001 | Transcutaneous ultrasound of the abdomen, with Doppler ultrasound of the digestive vessels |
| ZCQM005 | Transcutaneous ultrasound of the abdomen, with transcutaneous ultrasound of the pelvis |
| ZCQM004 | Transcutaneous ultrasound of the upper abdomen with ultrasound of the digestive vessels |
| ZCQM002 | Transcutaneous ultrasound of the abdomen, with transcutaneous ultrasound of the pelvis and Doppler ultrasound of the digestive vessels |

Codes used for cancer detection:

| ICD-10 codes | Diagnosis |
|---|---|
| C220 | Liver cell carcinoma |
| C221 | Intrahepatic bile duct carcinoma |

| | |
|---|---|
| C227 | Other specified carcinomas of liver |
| C229 | Malignant neoplasm of liver, not specified as primary or secondary |
| C240 | Malignant neoplasm of extrahepatic bile duct |
| C18 | Malignant neoplasm of colon |
| C** | Malignant neoplasm |
| D0* | Carcinoma in situ |

Codes used to analyze drug use:

| **ATC code** | **Label** |
|---|---|
| A05AA02 | Ursodeoxycholic acid (UDCA) |
| A02AA04 | Obeticholic acid |
| C10AB | Bezafibrate / fenofibrate |
| H02 | Corticosteroids |
| H02AB06 | Prednisolone |
| H02AB07 | Prednisone |
| A07EA06 | Budesonide |
| L04 | Immunosuppressive drug |
| L04AA06 | Mycophenolate Mofetil |
| L04AX01 | Azathioprine |
| L04AD02 | Tacrolimus |
| L04AD01 | Cyclosporine |

| **ATC code** | **Label** |
|---|---|
| A07EC02 | Mesalazine |
| L04AB01 | Etanercept |
| L04AB02 | Infliximab |
| L04AB04 | Adalimumab |
| L04AB05 | Certolizumab |
| L04AB06 | Golimumab |
| L04AA33 | Vedolizumab |
| J04AB02 | Rifampicin |

Codes used for the number of abdominal and liver scans performed during the first year after detection of the disease and for the six-monthly detection of cirrhosis:

| **CCAM code** | **Label** |
|---|---|

| | |
|---|---|
| ZCQM008 | Transcutaneous ultrasound of the abdomen |
| ZCQH001 | CT scan of the abdomen and pelvis, with intravenous injection of contrast medium |
| ZCQM002 | Transcutaneous ultrasound of the abdomen, with transcutaneous ultrasound of the pelvis and Doppler ultrasound of the digestive vessels |
| ZCQJ004 | Magnetic resonance imaging of the abdomen or pelvis, with intravenous injection of contrast medium |
| ZCQM010 | Transcutaneous ultrasound of the upper abdomen and pelvis |
| ZCQM004 | Transcutaneous ultrasound of the upper abdomen with Doppler ultrasound of the digestive vessels |
| ZCQM006 | Transcutaneous ultrasound of the upper abdomen |
| ZCQM001 | Transcutaneous ultrasound of the abdomen, with Doppler ultrasound of the digestive vessels |
| ZCQM011 | Transcutaneous ultrasound of the upper abdomen and pelvis and Doppler ultrasound of digestive vessels |
| ZCQN001 | Magnetic resonance imaging of the abdomen and pelvis, without intravenous injection of contrast medium |
| ZCQJ005 | Magnetic resonance imaging of the abdomen and pelvis, with intravenous injection of contrast medium |
| ZCQN002 | Magnetic resonance imaging of the abdomen or pelvis, without intravenous injection of contrast medium |
| ZCQK004 | CT scan of the abdomen and pelvis, without intravenous injection of contrast medium |
| ZCQK005 | CT scan of the abdomen or pelvis, without intravenous injection of contrast medium |
| ZCQM005 | Transcutaneous ultrasound of the abdomen, with transcutaneous ultrasound of the pelvis |
| ELQJ003 | Magnetic resonance imaging of vessels in the abdomen and/or pelvis, with magnetic resonance imaging of vessels in the limbs |
| ELQJ001 | Magnetic resonance imaging of vessels in the abdomen and/or pelvis |
| ELQH002 | CT scan of vessels in the abdomen and/or pelvis |
| ZCQH002 | CT scan of the abdomen or pelvis, with intravenous injection of contrast medium |
| ECQH011 | CT scan of vessels in the thorax and/or heart, with CT scan of vessels in the abdomen and/or pelvis |

| | |
|---|---|
| ZCQM005 | Transcutaneous ultrasound of the abdomen, with transcutaneous ultrasound of the pelvis |
| ELQH001 | Scan of liver vessels to study vascularisation at at least 3 different times |
| HLQM001 | Transcutaneous ultrasound of the liver and bile ducts |
| HLQM002 | Liver stiffness measurement using ultrasound pulse elastograph |

Codes used for the number of endoscopies and colonoscopies performed during the first year after detection of the disease:

| **CCAM code** | **Label** |
|---|---|
| HHQE | Colonoscopy |
| HEQE002 | Oeso-gastro-duodenal endoscopy |
| HMQA001 | Intraoperative endoscopy of the bile ducts, by transcystic approach or choledochotomy |
| HMQH003 | Retrograde cholangiography with infundibulotomy or pre-cutting of the major duodenal papilla, by oeso-gastro-duodenoscopy |
| HMQH007 | Retrograde cholangiography, by oeso-gastro-duodenoscopy |
| HMQH004 | Cholangiography, using transcutaneous injection of a contrast agent into the bile ducts, with ultrasound and/or radiological guidance. |
| HMQH006 | Cholangiography, by injection of contrast product into an external biliary drain |

Codes used for different types of hospitalization and complications of cirrhosis:

| **CCAM & ICD10 code** | **Procedure or diagnosis** |
|---|---|
| HLN HLC HLJ HLF HLS HLH | Hepato-biliary surgery |
| NBR_SUP_REA | Stays in intensive care |
| NBR_SUP_STF | Stays in intensive care unit |
| YYYY020 YYYY015 | Stays in intensive care (procedure) |
| Z511 | Chemotherapy session |
| HLNC003 HLNM001 HLNK001 HLNA007 HLNN900 | Hepatic tumour destruction by radiofrequency |
| Z944 | Liver transplantation (diagnosis) |

| | |
|---|---|
| HLEA001 HLEA002 HGEA002 HGEA004 | Liver transplantation (procedure) |
| HMQH HNQH001 HNQH003 | Retrograde cholangio-pancreatography |
| EHNE002 EHNE001 | Endoscopic ligation of oesophageal varices |
| EHCF002 | Transjugular intrahepatic portosystemic shunt (TIPS) |
| EDLF017 EDLF016 EDLL001 | Liver embolisation |
| R18 | Ascites |
| K922 | Digestive bleeding |
| G934 | Hepatic encephalopathy |
| K767 | Hepato-renal syndrome |
| J948 | Hydrothorax |
| I272 | Porto-pulmonary hypertension |
| C220 C221 C227 C229 C240 | Primary liver cancer |

Codes used for different types of work stoppage:
The variable used to calculate the number of days off work is PRS_ACT_NBR.

| **Code** | **Meaning** |
|---|---|
| 6110 | Normal daily allowances plus 6 months |
| 6111 | Normal daily allowances minus 3 months |
| 6112 | Normal daily allowances plus 3 months |
| 6113 | Reduced daily allowances minus 3 months |
| 6114 | Reduced daily allowances plus 3 months |
| 6115 | Increased daily allowances minus 3 months |
| 6116 | Increased daily allowances plus 3 months |
| 6117 | Partial daily allowances, loss of pay minus 3 months |
| 6118 | Partial daily allowances, loss of pay plus 3 months |
| 6119 | Increased daily allowances plus 6 months |
