# Supplementary material for: Epidemiology, comorbidities, treatments and outcomes of autoimmune liver diseases: A French nationwide study
Source: JHEP Rep. 2025 Aug 11;7(11):101546. doi: 10.1016/j.jhepr.2025.101546 (PMC12519275; doi:10.1016/j.jhepr.2025.101546)
Supplement: Multimedia component 4 [file mmc4.pdf]

Epidemiology, comorbidities, treatments and outcomes of autoimmune liver diseases: A French nationwide study

Authors

Christophe Corpechot, Pierre Hornus, Mallory Cals, …, Sara Lemoinne, Olivier Chazouillères, Angela Leburgue

Correspondence

christophe.corpechot@aphp.fr (C. Corpechot).

Graphical abstract

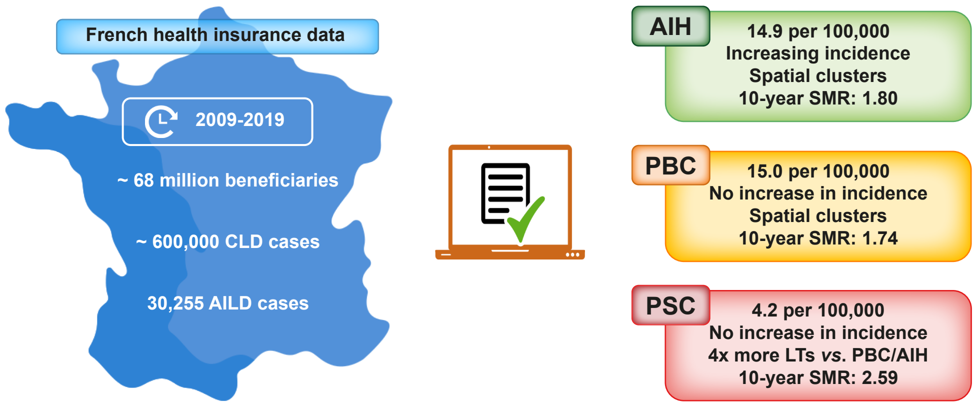

Highlights:

- Autoimmune hepatitis is the only autoimmune liver disease in France whose incidence is increasing.
- Autoimmune hepatitis and primary biliary cholangitis in France exhibit distinct geographic clustering.
- All autoimmune liver diseases in France confer a heightened risk of diabetes and malignancy.
- Despite current therapies, autoimmune liver diseases in France continue to exhibit elevated long-term mortality.
- Among autoimmune liver diseases in France, primary sclerosing cholangitis has the highest mortality and transplant rate.

Impact and implications:

The true burden of autoimmune liver diseases (AILDs) remains inadequately characterized. In this extensive study conducted in France, based on health insurance and hospital records collected between 2009 and 2019, we characterized the epidemiology, comorbidities, treatments and outcomes of autoimmune hepatitis, primary biliary cholangitis and primary sclerosing cholangitis. We confirm the rising incidence of AIH, the non-random geographic distribution of both autoimmune hepatitis and primary biliary cholangitis, the elevated risk of diabetes and cancer, and a persistently increased mortality across all AILDs – most notably in primary sclerosing cholangitis, where both excess mortality and the need for liver transplantation is higher than in other AILDs. These findings highlight the persistent gaps and unmet needs in the management of AILDs.

https://doi.org/10.1016/j.jhepr.2025.101546
© 2025 The Author(s). Published by Elsevier B.V. on behalf of European Association for the Study of the Liver (EASL). This is an open access article under the CC BY license (http://creativecommons.org/licenses/by/4.0/).

Epidemiology, comorbidities, treatments and outcomes of autoimmune liver diseases: A French nationwide study

Christophe Corpechot[1,2,*], Pierre Hornus[3], Mallory Cals[3,4], Pierre Rinder[3], Théo Marcille[3], Amina Malek[2], Karima Ben Belkacem[1], Farid Gaouar[1], Yasmina Chabane[1], Pierre-Antoine Corret[5], Paola Squarzoni[2], Pierre-Antoine Soret[1,2], Sara Lemoinne[1,2], Olivier Chazouillères[1,2], Angela Leburgue[5]

JHEP Reports **2025**. vol. 7 | 1–10

Check for updates

Abstract

Background & Aims: The epidemiology, clinical management, and prognosis of autoimmune liver diseases (AILDs) – including autoimmune hepatitis (AIH), primary biliary cholangitis (PBC), and primary sclerosing cholangitis (PSC) – vary according to geography and time. This study aimed to provide a comprehensive evaluation of the burden of AILDs in a Western European country.

Method: A nationwide retrospective study was performed using the French national health data system from 2009 to 2019. AIH and PBC were identified via ICD-10 codes, while PSC was defined using composite criteria. Prevalence, incidence, geographic patterns, comorbidities, treatments, liver transplant, and standardized mortality ratio were assessed.

Results: A total of 30,255 AILD cases were identified, representing 5% of chronic liver disease cases. The prevalence per 100,000 inhabitants was 14.9 for AIH, 15.0 for PBC, and 4.2 for PSC. Geographic variation was observed, with significant regional clustering of AIH and PBC cases. The incidence of AIH increased significantly over time, whereas that of PBC and PSC declined. Patients with AILDs exhibited higher rates of diabetes and all-cause malignancies compared to the general population. Ursodeoxycholic acid was underprescribed in PBC, while corticosteroids were frequently overused in both PBC and PSC, and ursodeoxycholic acid in AIH. Liver transplantation was performed four times more often in PSC than in either AIH or PBC. All AILDs were associated with elevated 10-year standardized mortality ratios: 1.80 for AIH, 1.74 for PBC, and 2.59 for PSC.

Conclusion: These findings confirm the rising incidence of AIH – but not PBC or PSC – the non-random geographic distribution of AIH and PBC, a higher risk of diabetes and cancer across all AILDs, and persistent excess mortality despite current treatment options.

© 2025 The Author(s). Published by Elsevier B.V. on behalf of European Association for the Study of the Liver (EASL). This is an open access article under the CC BY license (http://creativecommons.org/licenses/by/4.0/).

Introduction

Autoimmune liver diseases (AILDs) – a heterogeneous group primarily comprising autoimmune hepatitis (AIH), primary biliary cholangitis (PBC), and primary sclerosing cholangitis (PSC) – account for only 5% of all liver diseases but contribute disproportionately to the global burden due to their impact on quality of life, morbidity, mortality, and need for liver transplantation (LT).[1–4] The prevalence and incidence of AILDs vary by geographic region and time period; however, multiple studies indicate a steady rise in their incidence across Western countries.[5–8] At the same time, substantial progress in treating and managing AILDs, especially PBC and AIH, may have lessened their public health burden and unmet clinical needs over time.[9–12] In this context, the present study aimed to assess the contemporary burden of AIH, PBC, and PSC in a Western European country.

Patients and methods

Study design

We conducted a population-based observational longitudinal cohort study using data from the French National Health Data System (Système National des Données de Santé, SNDS) over the period from 1/12/2009 to 31/12/2019. The SNDS pools health insurance data (SNIIRAM database), hospital data (PMSI database), medical causes of death (Inserm's CépiDC database) and disability data (MDPH - CNSA database), as well as expenditure data on medical procedures (CCAM database) and medicinal products (Medic'AM database based on Anatomical Therapeutic Chemical [ATC] classification system) covered by the health insurance scheme. The SNDS registry encompasses the entire French population, including all beneficiaries of compulsory health insurance, regardless of

* Corresponding author. Address: Reference center for inflammatory biliary diseases and autoimmune hepatitis, Saint-Antoine Hospital, Assistance Publique – Hôpitaux de Paris; Inserm UMR_S938, Saint-Antoine Research Center, Sorbonne University; 184 rue du faubourg Saint-Antoine, 75571 Paris, Cedex 12, France.
E-mail address: christophe.corpechot@aphp.fr (C. Corpechot).
https://doi.org/10.1016/j.jhepr.2025.101546

their affiliation scheme – amounting to approximately 68.7 million individuals by the end of 2022.

The protocol was approved by the National health Insurance Fund (Caisse Nationale de l'Assurance Maladie [CNAM]), which is responsible for the SNDS, and the French Data Protection Committee (Commission Nationale de l'Informatique et des Libertés) under registration number *TPS-3403704*. The protocol, data extraction and analysis were supervised by Semeia© (Paris, France).

Case finding

Identification of AILD cases were based on the ICD-10, 2nd Edition codes found in health insurance and/or hospitalization records as the principal or associated diagnosis.[13] The ICD-10 diagnosis codes K754 and K743 were used to identify AIH and PBC, respectively. Due to the absence of a specific ICD-10 code for PSC during the period under investigation, PSC cases were defined as meeting the following three criteria: 1) presence of ICD-10 code K830 (cholangitis) as the principal or associated diagnosis, 2) presence of ICD-10 code K51 (ulcerative colitis) or K50 (Crohn disease) as the principal or associated diagnosis or a minimum of two occurrences of CCAM codes corresponding to hepatobiliary imaging (those codes are available in the appendix), 3) long-term treatment with ursodeoxycholic acid (UDCA), defined as a minimum of 3 purchases of the drug (ATC code A05AA02) over the study period (in France, UDCA is both approved and recommended for PSC patients, and the majority of these patients receive this treatment). For each case identified, we considered the inclusion date as the first appearance of the diagnosis during the study period. We excluded cases with no diagnosis or no indication of sex, those detected before or after the study period and those identified with ICD-10 code Z94.4 (LT status) before the study period.

Data collection

For each individual included in the study, age, sex and key comorbidities identified using CNAM algorithms were recorded at the time of inclusion. Data on consultations with hepatologists or gastroenterologists, hospital admissions within the year following inclusion, LT, deaths, and causes of death were collected throughout the follow-up period. The use of radiological examinations of the liver were also reported. Cirrhosis was identified in two ways: 1) using codes for cirrhosis or complications of cirrhosis (*i.e.* chronic liver failure, portal hypertension, esophageal or gastric varices, variceal bleeding, ascites, hydrothorax, hepatic encephalopathy, hepatorenal syndrome, hepatopulmonary syndrome, portopulmonary hypertension, or hepatocellular carcinoma) as a principle, related or associated diagnosis within 1 year following inclusion; 2) when liver imaging was repeated every 6 months for a minimum period of 2 years after inclusion. The number and frequency of most frequent comorbidities at inclusion, including active cancers, and those of medical consultations, hospitalizations and work stoppages during the year following inclusion were assessed. We used ATC codes to assess drug use in the year after inclusion for patients aged over 18 years for whom a full year of data was available. The following drugs of interest were the focus of our study: systemic corticosteroids (prednisone, prednisolone, budesonide), non-corticosteroid immunosuppressive drugs (azathioprine, mycophenolate mofetil, tacrolimus, ciclosporin), UDCA, obeticholic acid and fibrates (bezafibrate, fenofibrate). Associated drugs of interest included: 5-aminosalicylates, TNF-α inhibitors, integrin inhibitors and rifampicin. Year-, age-, and sex-specific population estimates were retrieved from the French National Institute for Statistics and Economic Studies (INSEE, www.insee.fr).[14] All the codes (ICD-10, CCAM, ATC) used in this study are available in the Appendix.

Statistical analysis

Prevalence rates per 100,000 people were calculated for each French department, as well as for metropolitan France overall. These rates were determined at the end of the observation period, considering all patients who were alive at that time. As it was not possible to formally exclude the possibility that some prevalent patients may not have been identified during this 10-year period, these prevalence rates should be regarded as minimum estimates of actual rates. Overall and departmental incidence rates per 100,000 people were calculated on a quarterly basis throughout the study period, separating the adult population (≥18 years) from the pediatric population (<18 years). Incidence rates were standardized based on official data from the general population, adjusted for age, sex, and study period. The total number of individuals recorded with chronic liver disease in France at the end of the study period was obtained from publicly available official SNDS data. The correlation between incidence rate and time was assessed using simple linear regression. The prevalence rates of the main comorbidities identified by the CNAM algorithms were estimated at the date of inclusion and compared with the rates expected in the general population after weighting by age and sex. Spatial cluster analysis was performed by testing the autocorrelation of departmental prevalence rates over 10 years using the local Moran's I index and a Moran scatter plot.[15] A correlation analysis with the departmental density of hepato-gastroenterologists was performed to assess possible diagnosis bias. Alluvial diagrams were used to assess changes in drug use during the year following inclusion. A Kaplan-Meier analysis was performed to estimate cumulative survival rates and corresponding 95% CIs. Date of diagnosis was used as the starting point of the disease. Both overall and transplant-free survival were estimated. Standardized mortality ratio and the corresponding CIs were calculated as the ratios of observed to expected deaths in the study cohort. The expected number of deaths was calculated based on mortality by age and gender in the general population. The 10-year LT incidence rates and 95% CIs were calculated using a cumulative incidence function accounting for competing risk of death, according to the method of Kalbfleisch and Prentice.[16] To test the hypothesis that mortality is primarily increased in the first year after diagnosis due to late diagnosis of severe forms of the disease, as severe acute AIH, decompensated PBC or PSC revealed by cholangiocarcinoma, a sensitivity analysis was performed by excluding patients whose follow-up after diagnosis was limited to 1 year or less, *i.e.* using 1 year after diagnosis as a landmark time. Quantitative data was expressed as mean and SD and qualitative data as number

and percentage. Statistical analyses were performed using R version 4.1.3 (R Foundation for Statistical Computing, Vienna, Austria). p <0.05 was considered statistically significant.

Results

Patient characteristics

In total, out of approximately 68.7 million people covered by the health insurance scheme during the study period (2009-2019), 14,124 people met the diagnostic criteria for PBC, 12,616 for AIH and 3,515 for PSC, making a total of 30,255 patients (Fig. S1). Over the same period, around 600,000 patients were recorded as suffering from chronic liver disease, thus bringing the relative prevalence of AILDs within all reported cases of chronic liver disease to 5%. Approximately 15% of patients with AILD received supplementary universal health cover, compared to 8% in the general population. Table 1 presents the demographics and baseline characteristics of the three AILD cohorts at diagnosis. The age distribution profiles at diagnosis are shown in Fig. 1. The mean age (SD) at presentation was 53.6 (21.2) years for AIH, 61.6 (17.7) years for PBC, and 43.7 (21.1) years for PSC. The proportion of patients diagnosed before the age of 18 was 10.8% for PSC, 6.9% for AIH and 2.3% for PBC. The overall percentages of females at diagnosis were 74.5% for PBC, 72.9% for AIH, and 49.9% for PSC. A total of 1,224 (4.6%) of the 26,740 patients diagnosed with PBC or AIH had overlapping diagnostic criteria for both diseases. Similarly, 669 (4.1%) of the 17,639 patients diagnosed with PSC or AIH had diagnostic criteria common to both diseases. The percentage of patients with PSC diagnosed with inflammatory bowel disease (IBD) was 61.7%. The proportion of patients with at least one extrahepatic autoimmune condition (excluding IBD) was 9.5% in AIH, 8.7% in PBC, and 6.2% in PSC. Table S1 details the most frequently identified extrahepatic autoimmune conditions at the time of AILD diagnosis. Cirrhosis was identified at diagnosis in 11.8% of cases based on related diagnostic codes (PBC 13.4%, AIH 10.8%, PSC 9.3%), and in 11.9% when inferred from the frequency of liver imaging (PBC 10.2%, AIH 9.9%, PSC 25.5%). This latest estimate of cirrhosis prevalence in PSC is biased by the diagnostic algorithm used, which involves repeated hepatobiliary imaging tests. Among patients with cirrhosis at the time of diagnosis, the mean age was 59.7 years for AIH, 63.2 years for PBC, and 48.2 years for PSC. In patients younger than 18 years, the proportion diagnosed with cirrhosis and the corresponding mean age at diagnosis were 5.1% and 12.6 years for AIH, and 11.6% and 9.4 years for PSC, respectively. PBC data are not presented due to uncertainty about the diagnostic accuracy in this age group.

Prevalence and geographical distribution

The national prevalence rate per 100,000 people was 15.0 for PBC, 14.0 for AIH and 4.2 for PSC (Table 2). For each type of AILD, prevalence varied greatly depending on the geographical area (Fig. 2). Clearly, AIH appeared to be more common in the south-west, while PBC was more common in the east of the country. The highest departmental prevalence rates per 100,000 people were 35.2 for PBC, 27.6 for AIH and 10.5 for PSC. Local Moran-I statistic indicated significant positive spatial autocorrelation of cases (*i.e.* the presence of clusters) for PBC (Moran-I index 0.30, p <0.001) and AIH (Moran-I index 0.28, p <0.001), but not for PSC – despite a trend towards statistical autocorrelation (Moran-I index 0.11, p = 0.062) (Fig. S2). No correlation was found between departmental prevalence and local density of practitioners (Fig. S3).

Average and time-dependent incidence

The overall average incidence rate per 100,000 person-years was 2.2 for PBC, 1.9 for AIH and 0.7 for PSC (Table 2). The quarterly incidence rate was assessed over time for each AILD, distinguishing between the population aged over and under 18 and taking into account changes in the French population over the period studied. The incidence of AIH increased significantly in both adults (+0.012/100,000 person-years, p <0.01) and young people (+0.010/100,000 person-years, p <0.01), while that of PBC decreased in both age categories (-0.010/100,000 person-years, p = 0.04, and -0.002/100,000 person-years, p <0.01, respectively) and that of PSC decreased in adults (-0.006/100,000 person-years, p = 0.04) but remained unchanged in young people (+0.001/100,000 person-years, p = 0.68) (Fig. 3).

Comorbidities, consultations, hospitalizations and medical leave

The prevalence of the main comorbidities identified by the CNAM algorithms at inclusion was compared with that of the general population for each AILD (Fig. 4). Diabetes and active cancers were found to be more common at inclusion in the three AILDs than in the general population. Other comorbidities were broadly comparable. Primary liver cancer (*i.e.* hepatocellular or cholangiocellular carcinomas) was present at inclusion in 0.8% of patients with AIH, 1.8% with PBC and 1.4% with PSC. The 10 most frequently reported active cancer codes in the year prior to inclusion are reported in Table S2.

The median (IQR) number of consultations with a hepatologist or a gastroenterologist in the year following diagnosis was 3 (2–5) for AIH, 3 (2–4) for PBC and 3 (2–5) for PSC (Fig. S4). The median (IQR) number of hospital admissions in

Table 1. Demographics and baseline characteristics of AILD populations at diagnosis.

Characteristic	AIH (n = 12,616)	PBC (n = 14,124)	PSC (n = 3,515)
Mean age (years, ± SD)	53.6 ± 21.2	61.6 ± 17.7	43.7 ± 21.1
Female (%)	72.9%	74.5%	49.9%
<18 years (%)	6.9%	2.3%	10.8%
Cirrhosis (%)[†]	10.8%	13.4%	9.3%
IBD (%)	1.3%	1.0%	61.7%
Extrahepatic AID (%)[‡]	9.5%	8.7%	6.2%

AI(L)D, autoimmune (liver) disease; AIH, autoimmune hepatitis; IBD, inflammatory bowel disease; PBC, primary biliary cholangitis; PSC, primary sclerosing cholangitis.
[†]Based on related diagnostic codes.
[‡]Excluding IBD.

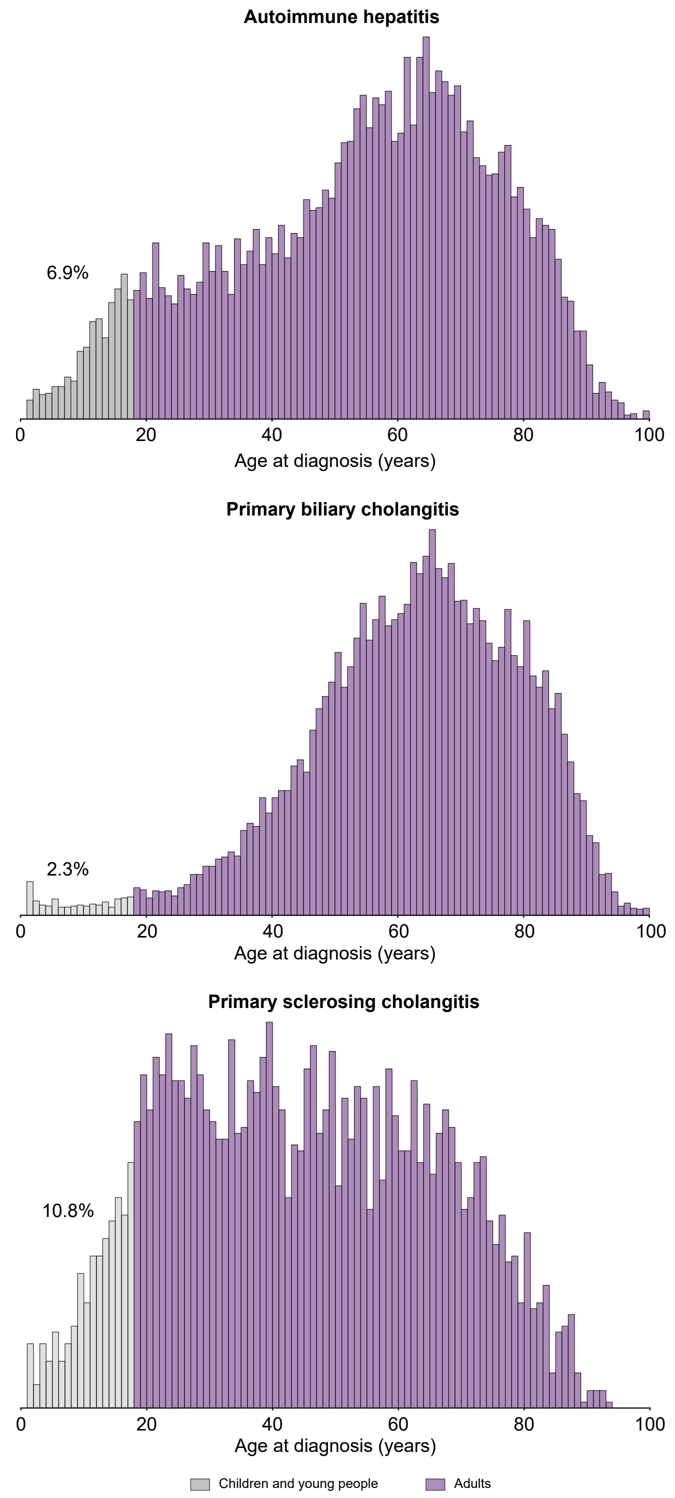

Fig. 1. Age distributions of AILDs at inclusion in the cohort. The overall percentage of children and young people (light grey columns) is shown. AILD, autoimmune liver disease.

the year following diagnosis was 2 (1–3) for AIH, 1 (1–3) for PBC and 3 (2–5) for PSC (Fig. S5), while the median (IQR) number of inpatient days in the year following diagnosis was 5 (2–13) for AIH, 2 (1–9) for PBC and 7 (2–15) for PSC (Fig. S6).

Table 2. Prevalence and incidence of AILDs.

Measure	AIH	PBC	PSC
Prevalence rate (per 100,000 population)	14.0	15.0	4.2
Incidence rate (per 100,000 per year)	1.9	2.2	0.7

AIH, autoimmune hepatitis; AILD, autoimmune liver disease; IBD, inflammatory bowel disease; PBC, primary biliary cholangitis; PSC, primary sclerosing cholangitis. Prevalence estimates were derived as of the final year of the study (2019), whereas incidence rates were calculated over the full study period (2009–2019).

The percentage of work stoppage of at least 1 day (median duration) in the year following inclusion in the cohort for the population aged over 18 and under 60 was 28% (27 days) for AIH, 22% (18 days) for PBC and 30% (16 days) for PSC. Medical leave of more than 1 year was observed in approximately 5% of patients regardless of AILD.

Medication use

The three treatments of interest most frequently dispensed in the year following inclusion in patients over 18 years of age were non-corticosteroid immunosuppressive drugs (59.9%), corticosteroids (59.7%) and UDCA (31.2%) for AIH, UDCA (72.8%), corticosteroids (33.8%) and non-corticosteroid immunosuppressive drugs (11.7%) for PBC, and UDCA (89.1%), corticosteroids (43.6%) and non-corticosteroid immunosuppressive drugs (35.7%) for PSC (Table S3). While associated drugs of interest were dispensed anecdotally for AIH and PBC, not surprisingly 5-aminosalicylates (27.8%) and TNF-α inhibitors (12.6%) were commonly delivered in the context of PSC. Overall, treatments for the three AILDs remained stable during the year following inclusion, except in AIH, where the number of patients receiving combined corticosteroid and non-corticosteroid immunosuppressive therapy decreased in favor of those receiving non-corticosteroid immunosuppressive therapy alone (Fig. S7).

Liver transplant, death and cause of death

The 5- and 10-year overall survival rates (95% CIs) after inclusion in the cohort for the population aged over 18 were 86% (85%–87%) and 74% (72%–76%) for AIH, 79% (78%–80%) and 66% (65%–68%) for PBC, and 87% (86%–89%) and 76% (73%–78%) for PSC (Fig. 5). The corresponding transplant-free survival rates were 84% (83%–85%) and 72% (70%–74%) for AIH, 78% (77%–79%) and 64% (63%–66%) for PBC, and 79% (77%–80%) and 64% (61%–67%) for PSC. Cumulative 10-year incidence rates (95% CI) for LT were 4% (3%–4%) for PBC, 4% (3%–4%) for AIH, and 16% (14%–17%) for PSC. Excluding patients whose follow-up since diagnosis was ≤1 year did not significantly alter the results, confirming the validity of long-term survival rates (Table S4).

The 5- and 10-year overall survival rates in an age- and sex-matched standardized control population over 18 years of age were 94% and 86% for AIH, 91% and 81% for PBC, and 96% and 92% for PSC (Fig. 5). The 10-year standardized mortality ratio (95% CI) quantifying the increase in the mortality of the study cohort with respect to the general population was 1.80 (1.68–1.94) for AIH, 1.74 (1.66–1.83) for PBC, and 2.59 (2.30–2.91) for PSC.

Data on the cause of death were available for 48%, 50% and 45% of patients who died of AIH, PBC and PSC, respectively. The 10 most frequently reported diagnoses

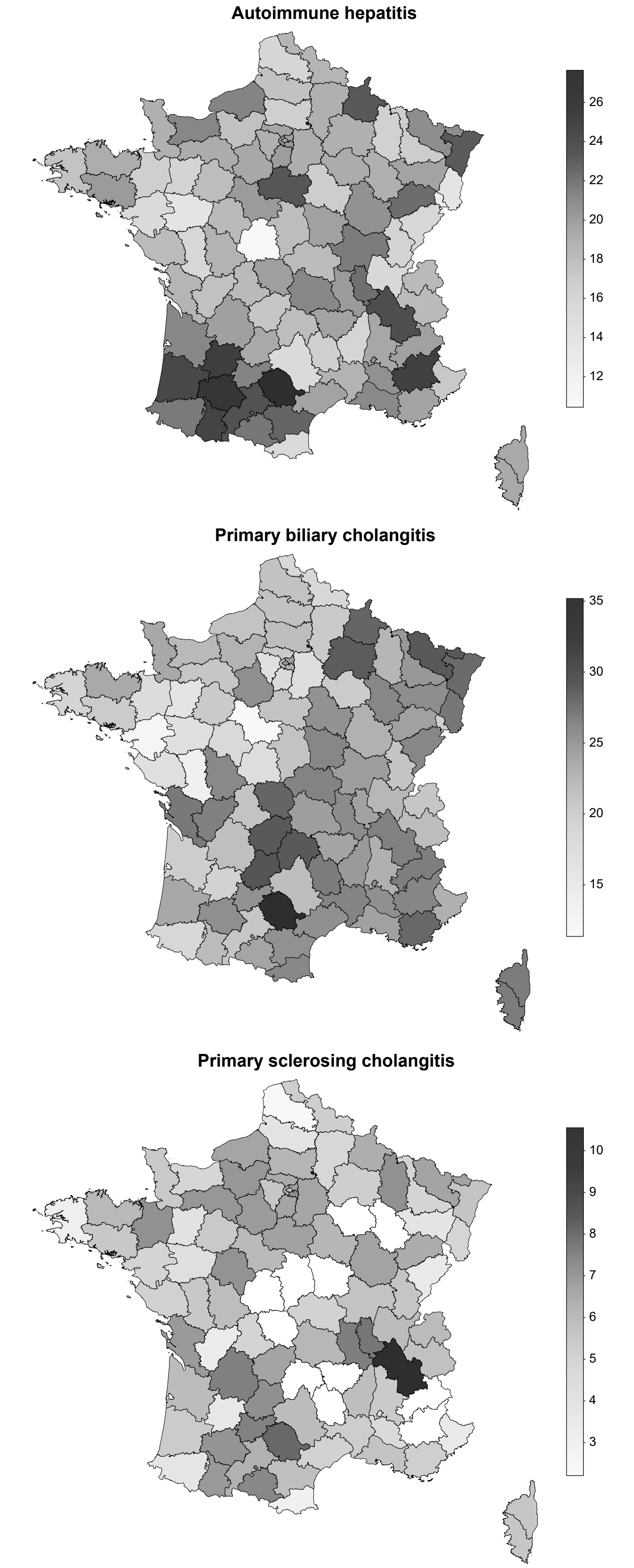

Fig. 2. Mapping of prevalence rates of AILDs by department. The prevalence scale is expressed as the number of cases per 100,000 people. AILD, autoimmune liver disease.

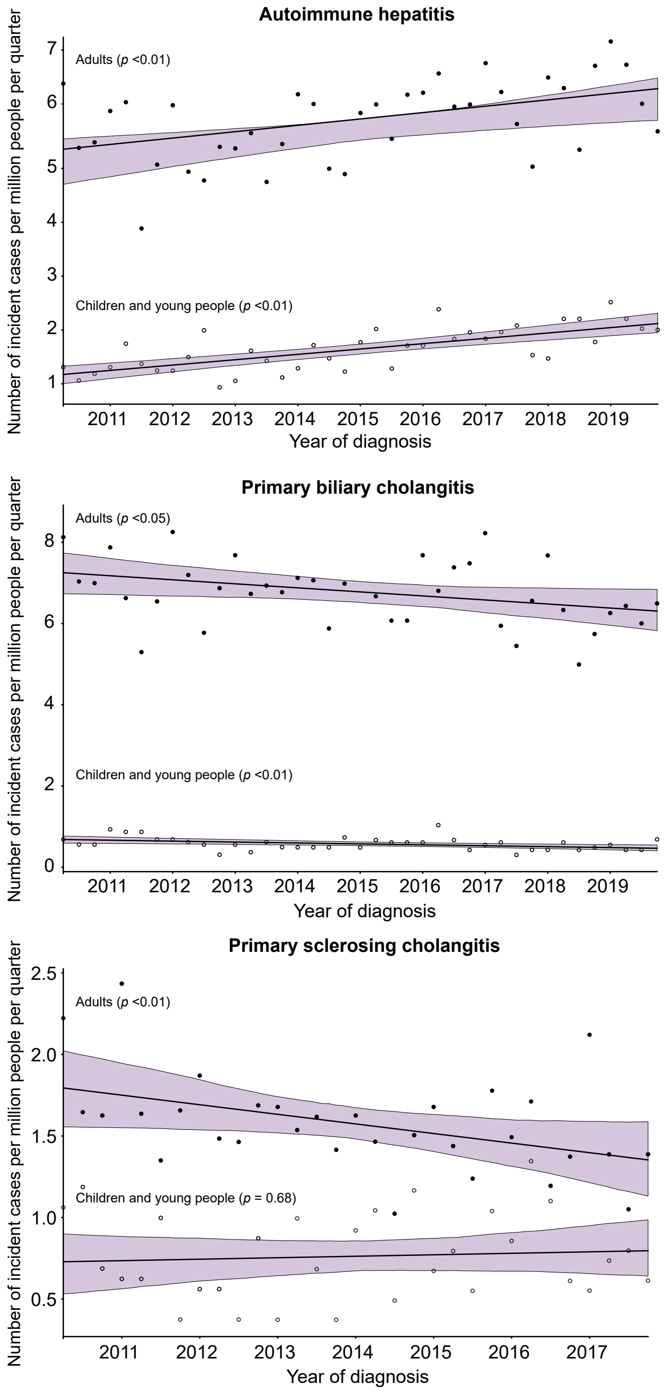

Fig. 3. Incidence rate of AILDs as a function of time. Quarterly incidence rates and corresponding linear trend lines are presented separately for the adult and child/adolescent populations. *P* values are derived from Pearson's correlation coefficient. AILDs, autoimmune liver diseases.

associated with death for each AILD are shown in Table S5. Of these, liver failure and sepsis were reported in approximately 15% and 10% of patients, respectively, independent of AILD, while cholangiocellular carcinoma was reported in 16% of patients with PSC and hepatocellular carcinoma in 10% of patients with PBC.

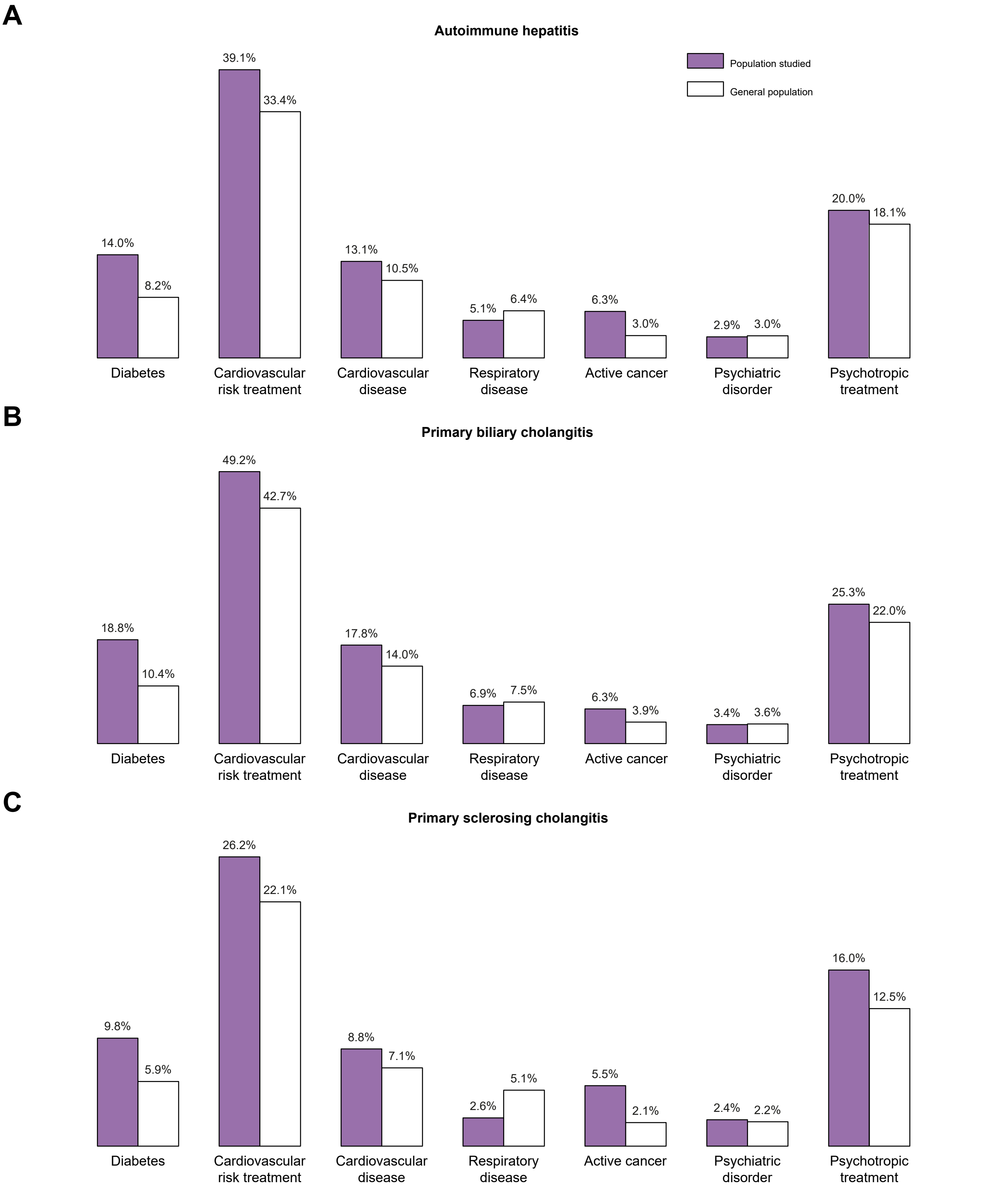

Fig. 4. Distribution of the main comorbidities identified by the CNAM algorithm. AILDs are shown in grey, while the general population is shown in white. AILDs, autoimmune liver diseases; CNAM, Caisse Nationale de l'Assurance Maladie.

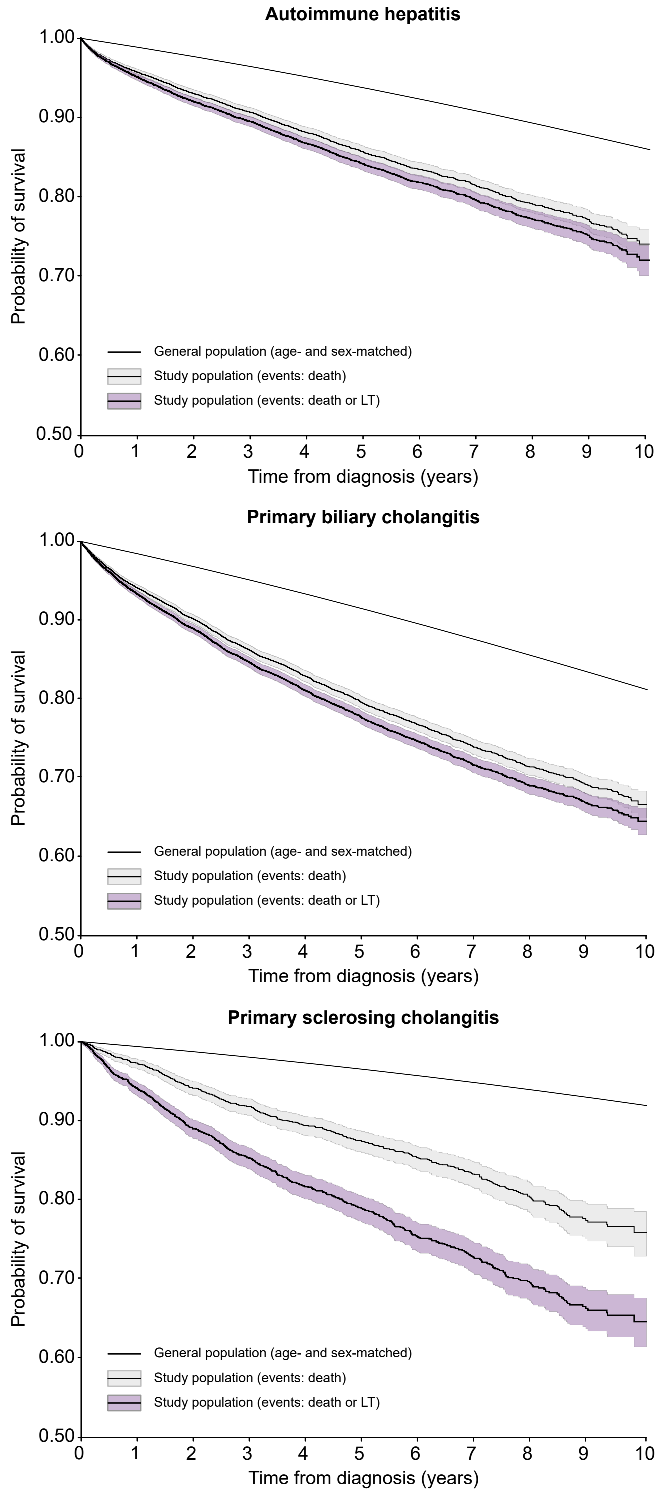

Fig. 5. Overall survival rate and LT-free survival rate of AILDs as a function of time. Overall survival rates (95% CIs) are represented by the medium thickness curve (light grey area), while LT-free survival rates (95% CI) are represented by the thick curve (dark grey area). Overall survival rates for the general population matched for age and sex are represented by the thin curve. For better visibility, the y-axis is cut at 0.50. AILDs, autoimmune liver diseases; LT, liver transplantation.

Discussion

The aim of this study was to systematically assess the current burden of autoimmune liver diseases (AILDs) in a representative Western European country (*i.e.* France) by analyzing health insurance data. The data used in this study were sourced from the French National Health Data System (SNDS) and encompass 30,255 patients identified between 2009 and 2019, drawn from a population of approximately 68 million insured individuals. The extensive size, comprehensive coverage, depth of detail, and minimal bias of the SNDS data make it an unparalleled resource for advancing understanding of these diseases. Improved knowledge of the true prevalence of AILDs and patients' care pathways will enable practitioners to identify deviations from clinical guidelines and prioritize areas for diagnosis and patient support.

The average prevalence rates of PBC, AIH, and PSC estimated in this study align closely with those reported in recent Western European research.[5,17,18] PBC and AIH exhibit similar prevalence rates, each affecting approximately 15 per 100,000 individuals, while PSC is three to four times less common, with a prevalence of about 4 per 100,000. As expected, PBC and AIH predominantly affect women, whereas PSC shows an approximately equal distribution between males and females. The relatively low proportion of women with PBC (75%) aligns with recent studies suggesting that the percentage of men with PBC has been underestimated, particularly in Southern Europe, where female-to-male ratios can be as low as 2.3:1.[19] Similarly, the relatively advanced age at diagnosis for patients with PBC (62 years) supports the trend that PBC is increasingly being identified in older individuals.[20] The fact that PBC was reported anecdotally before the age of 18, while AIH and PSC were identified before this age in 7% and 11% of cases, respectively, is also consistent with the known epidemiology of these diseases. Approximately 5% of patients diagnosed with PBC or PSC also met the diagnostic criteria for AIH, consistent with what is commonly reported in the literature.[21] Finally, suspected cirrhosis was diagnosed in 12% of patients with AILDs, irrespective of the diagnostic approach, aligning with anticipated results.

Our results indicate that the incidence of AIH in France increased significantly between 2009 and 2019, whereas the incidence of PBC and PSC declined during the same period. This rising trend in AIH incidence is consistent with observations reported globally.[6,8,22–24] While a diagnostic bias – potentially driven by the increased use of autoantibody testing in routine clinical practice – cannot be excluded, the substantial rise in autoimmunity and autoimmune diseases worldwide is likely linked to changes in environmental exposures.[25] In this context, AIH is likely the most prototypical autoimmune disease among the AILDs, whereas PBC and PSC may be more accurately described as immune-mediated conditions rather than classic primary autoimmune diseases. Our findings align well with this broader understanding.

The literature on the incidence of PBC and PSC is mixed, with some studies reporting an increase, while many others suggest that incidence rates have remained generally stable.[5,8,24,26–28] Our results even suggest that it may have declined in the last 15 years, albeit with a low level of statistical

significance. To our knowledge, this is the first time that a potential decline in these presumed autoimmune cholangiopathies has been reported. Importantly, the severity of PBC has been shown to decline gradually over recent decades.[20] While this apparent shift in the disease's natural history may partly reflect earlier diagnosis, it could also indicate a true reduction in disease severity, potentially driven by changes in environmental and/or genetic risk factors. Similar changes in the clinical presentation of PSC have not been observed. However, studies over the past 20 years, particularly in Scandinavian countries, have reported a decline in the incidence of IBD, including ulcerative colitis and Crohn's disease, which may partly account for a corresponding decrease in PSC incidence.[29]

Our findings reveal a non-random geographical distribution of AILDs, with significant spatial clusters identified for both AIH and PBC across the country. This pattern is unlikely to be attributable to diagnostic bias, as no correlation was observed with healthcare practitioner density. Latitude has been proposed as a risk factor for AILDs due to its influence on vitamin D status, which is itself associated with the risk of developing autoimmune diseases.[30] An association between more northerly latitude and the incidence of PBC and AIH has been reported, while an ascending gradient from east to west and south to north has been described for the prevalence of PSC.[31,32] Our findings do not support these previous observations, instead revealing a higher prevalence of AIH in southern France and PBC in the eastern regions, while PSC appeared evenly distributed. Nevertheless, these data further support the hypothesis that environmental and/or genetic factors contribute to the pathogenesis of these diseases.

The prevalence of diabetes and all-cause active cancers was 1.5 to 2 times higher in individuals with AILDs compared to a matched general population, consistent with findings in the existing literature. Both type 1 and type 2 diabetes have been associated with AILDs, either as predisposing factors or as potential consequences of the diseases themselves or their treatments.[33–36] AILDs are known to predispose individuals to primary hepatobiliary cancers and, in the case of PSC, also to colorectal, gastric, and pancreatic malignancies. Our findings are consistent with this association, and the observed increase in the prevalence of all-cause cancers is therefore not unexpected. Extra-digestive malignancies may also be more common in patients with AILDs – particularly skin cancers and lymphomas in AIH, as well as breast and bladder cancers in PBC.[37–41] Conversely, immune-mediated liver injury caused by checkpoint inhibitors used in cancer immunotherapy may also contribute to this association.[42,43]

The reported medication dispensing generally aligned with treatment guidelines for each AILD. However, several unexpected findings warrant attention. Firstly, UDCA was dispensed to only 75% of patients with PBC within 1 year of diagnosis. This aligns with recent studies indicating suboptimal UDCA use in real-world PBC populations, even in Western countries such as the US – where only 70% of patients receive this therapy – and Germany.[24,44] This may reflect a lack of awareness about the necessity of treatment, especially in elderly patients, or poor adherence on the part of patients. It may also be attributable to a high proportion of mild PBC cases (characterized by only slight elevations in serum liver enzymes) that are managed with monitoring rather than immediate treatment.[45,46] Secondly, an unexpectedly high rate of corticosteroid use was observed in patients with PBC and PSC, affecting approximately one-third of cases. The indications for this treatment were not specified and it remains unclear whether they were related to cholangiopathy (with or without features of AIH), to IBD in PSC, or to a concurrent extrahepatic autoimmune disease associated with either cholangiopathy. Finally, UDCA was prescribed to approximately one-third of patients with AIH, either as an adjunct therapy or even as monotherapy. This unexpectedly frequent use of UDCA in AIH – exceeding the expected rate of overlap syndromes – may reflect specific prescribing practices, particularly among pediatricians due to the higher prevalence of overlap syndrome in children, as well as the recognized beneficial effects of UDCA in managing AIH.[47,48] This observation is not reported in the recent study of the European Reference Network registry, although it remains unclear whether this treatment was specifically examined.[49]

Survival rates for each AILD generally aligned with those reported in recent cohorts, except for PBC, where 5- and 10-year liver transplant-free survival rates were approximately 10% lower than typically observed.[50–52] This difference may be attributable to the older age of our study population at inclusion (62 years) compared to cohorts from tertiary centers, where the average age is typically around 50 years. The need for LT was four times higher for PSC than PBC or AIH, reflecting both the lack of effective treatments and the more aggressive natural course of PSC. Even after accounting for this difference, the standardized mortality ratio for PSC remained significantly higher than that for PBC or AIH, establishing PSC as the most lethal AILD to date. Nevertheless, PBC and AIH also demonstrated excess mortality compared to the general population, highlighting ongoing unmet clinical needs in these diseases.

Our study has several limitations. Since it relied solely on health insurance records, we cannot entirely exclude the possibility that some patients with AILD were not identified. However, this proportion is likely very small, as AILDs require long-term, often costly treatment and follow-up, typically necessitating specific social care. Additionally, diagnoses were based solely on reported codes, without access to individual clinical data – such as serologies, imaging, or histology – to verify accuracy. For example, the 2% rate of 'pediatric PBC' highlights the inherent margin of error in such administrative databases and may serve as an indirect indicator of data quality. Moreover, because there was no specific ICD-10 code for PSC during the study period, we used composite criteria to identify patients with PSC, which could introduce diagnostic heterogeneity. Nonetheless, the demographic profile and prevalence of characteristic comorbidities (IBD, AIH) in this group align with typical PSC populations, supporting the validity of our approach. Finally, the absence of clinical, biological, and radiological data limited our ability to accurately characterize patient populations, particularly regarding disease severity at diagnosis.

In conclusion, this population-based study confirms the rising incidence of AIH – but not of PBC or PSC – the non-random geographical distribution of AIH and PBC, the increased risk of diabetes and all-cause cancers, and the persistence of excess mortality across all AILDs despite current treatments. While UDCA appears to be suboptimally

prescribed in PBC, corticosteroids are used more frequently than expected in both PBC and PSC, and UDCA is also commonly administered in AIH. Future research should explore potential regional variations in treatment patterns, liver-related mortality, and LT rates, and investigate whether these differences correlate with disease prevalence.

Affiliations

[1]*Reference Center for Inflammatory Biliary Diseases and Autoimmune Hepatitis, European Reference Network (ERN) on Hepatological Diseases Rare-Liver, INSERM UMR_S938, Saint-Antoine Hospital, Assistance Publique - Hôpitaux de Paris, Sorbonne University, Paris, France;* [2]*French Network for Rare Liver Disease in Children and Adults FILFOIE, Saint-Antoine Hospital, Assistance Publique - Hôpitaux de Paris, Paris, France;* [3]*Semeia©, Paris, France;* [4]*Heva©, Lyon, France;* [5]*ALBI Patient Association, Versailles, France*

Abbreviations

AIH, autoimmune hepatitis; AILD, autoimmune liver disease; ATC, Anatomical Therapeutic Chemical; HR, hazard ratio; LT, liver transplantation; PBC, primary biliary cholangitis; PSC, primary sclerosing cholangitis; UDCA, ursodeoxycholic acid.

Financial support

This study was supported by a research grant from the French rare liver disease network FILFOIE and a grant from the patient organization ALBI.

Conflict of interest

The authors of this study declare that they do not have any conflict of interest.

Please refer to the accompanying ICMJE disclosure forms for further details.

Authors' contributions

CC and AL contributed to the design, implementation, and supervision of the study. CC also participated in the analysis and interpretation of the results and wrote the manuscript. PH, MC, PR, and TM contributed to the protocol design, submitted it to the French authorities (CNAM and CNIL), and handled data import and analysis from the SNDS. AM, KBB, FG, YC, PC, PS, PAS, SL, and OC reviewed and approved the final manuscript.

Data availability

The data that support the findings of this study are available from the corresponding author upon reasonable request. The data are not publicly available due to privacy or ethical restrictions.

Supplementary data

Supplementary data to this article can be found online at https://doi.org/10.1016/j.jhepr.2025.101546.

References

Author names in bold designate shared co-first authorship

[1] Decock S, McGee P, Hirschfield GM. Autoimmune liver disease for the non-specialist. BMJ 2009;339:b3305.
[2] Heneghan MA, Yeoman AD, Verma S, et al. Autoimmune hepatitis. Lancet 2013;382:1433–1444.
[3] Dyson JK, Beuers U, Jones DEJ, et al. Primary sclerosing cholangitis. Lancet 2018;391:2547–2559.
[4] **Tanaka A**, **Ma X**, Takahashi A, et al. Primary biliary cholangitis. Lancet 2024;404:1053–1066.
[5] **de Veer RC**, **van Hooff MCB**, Werner E, et al. Incidence and prevalence of primary biliary cholangitis in The Netherlands - a nationwide cohort study. JHEP Rep 2024;6:101132.
[6] Hahn JW, Yang HR, Moon JS, et al. Global incidence and prevalence of autoimmune hepatitis, 1970-2022: a systematic review and meta-analysis. EClinicalMedicine 2023;65:102280.
[7] Mehta TI, Weissman S, Fung BM, et al. Global incidence, prevalence and features of primary sclerosing cholangitis: a systematic review and meta-analysis. Liver Int 2021;41:2418–2426.
[8] Lamba M, Ngu JH, Stedman CAM. Trends in incidence of autoimmune liver diseases and increasing incidence of autoimmune hepatitis. Clin Gastroenterol Hepatol 2021;19:573–579 e571.
[9] Poupon RE, Balkau B, Eschwege E, et al. A multicenter, controlled trial of ursodiol for the treatment of primary biliary cirrhosis. N Engl J Med 1991;324:1548–1554.
[10] Nevens F, Andreone P, Mazzella G, et al. A placebo-controlled trial of obeticholic acid in primary biliary cholangitis. N Engl J Med 2016;375:631–643.
[11] Corpechot C, Chazouilleres O, Rousseau A, et al. A placebo-controlled trial of bezafibrate in primary biliary cholangitis. N Engl J Med 2018;378:2171–2181.
[12] Johnson PJ, McFarlane IG, Williams R. Azathioprine for long-term maintenance of remission in autoimmune hepatitis. N Engl J Med 1995;333:958–963.
[13] World Health Organization. In: ICD-10: International statistical classification of diseases and related health problems. 10th revision, 2nd ed. Geneva: World Health Organization; 2004.
[14] Bellamy V, Beaumel C. Bilan démographique 2015: Le nombre de décès au plus haut depuis l'après-guerre. Insee Première 2016:1581.
[15] Chen Y. Spatial autocorrelation equation based on Moran's index. Sci Rep 2023;13:19296.
[16] Prentice RL, Kalbfleisch JD, Peterson Jr AV, et al. The analysis of failure times in the presence of competing risks. Biometrics 1978;34:541–554.
[17] **Lv T**, **Li M**, Zeng N, et al. Systematic review and meta-analysis on the incidence and prevalence of autoimmune hepatitis in Asian, European, and American population. J Gastroenterol Hepatol 2019;34:1676–1684.
[18] Liang H, Manne S, Shick J, et al. Incidence, prevalence, and natural history of primary sclerosing cholangitis in the United Kingdom. Medicine 2017;96:e7116.
[19] Lleo A, Jepsen P, Morenghi E, et al. Evolving trends in female to male incidence and male mortality of primary biliary cholangitis. Sci Rep 2016;6:25906.
[20] Murillo Perez CF, Goet JC, Lammers WJ, et al. Milder disease stage in patients with primary biliary cholangitis over a 44-year period: a changing natural history. Hepatology 2018;67:1920–1930.
[21] Trivedi PJ, Hirschfield GM. Review article: overlap syndromes and autoimmune liver disease. Aliment Pharmacol Ther 2012;36:517–533.
[22] Takahashi A, Ohira H, Abe K, et al. Increasing incidence of acute autoimmune hepatitis: a nationwide survey in Japan. Sci Rep 2020;10:14250.
[23] Gronbaek L, Otete H, Ban L, et al. Incidence, prevalence and mortality of autoimmune hepatitis in England 1997-2015. A population-based cohort study. Liver Int 2020;40:1634–1644.
[24] Sebode M, Kloppenburg A, Aigner A, et al. Population-based study of autoimmune hepatitis and primary biliary cholangitis in Germany: rising prevalences based on ICD codes, yet deficits in medical treatment. Z Gastroenterol 2020;58:431–438.
[25] Miller FW. The increasing prevalence of autoimmunity and autoimmune diseases: an urgent call to action for improved understanding, diagnosis, treatment, and prevention. Curr Opin Immunol 2023;80:102266.
[26] Marschall HU, Henriksson I, Lindberg S, et al. Incidence, prevalence, and outcome of primary biliary cholangitis in a nationwide Swedish population-based cohort. Sci Rep 2019;9:11525.
[27] McNally RJ, James PW, Ducker S, et al. No rise in incidence but geographical heterogeneity in the occurrence of primary biliary cirrhosis in North East England. Am J Epidemiol 2014;179:492–498.
[28] Lindkvist B, Benito de Valle M, Gullberg B, et al. Incidence and prevalence of primary sclerosing cholangitis in a defined adult population in Sweden. Hepatology 2010;52:571–577.
[29] Forss A, Clements M, Bergman D, et al. A nationwide cohort study of the incidence of inflammatory bowel disease in Sweden from 1990 to 2014. Aliment Pharmacol Ther 2022;55:691–699.
[30] Dankers W, Colin EM, van Hamburg JP, et al. Vitamin D in autoimmunity: molecular mechanisms and therapeutic potential. Front Immunol 2016;7:697.
[31] Webb GJ, Ryan RP, Marshall TP, et al. The epidemiology of UK autoimmune liver disease varies with geographic latitude. Clin Gastroenterol Hepatol 2021;19:2587–2596.

[32] Saffioti F, Mavroeidis VK. Review of incidence and outcomes of treatment of cholangiocarcinoma in patients with primary sclerosing cholangitis. World J Gastrointest Oncol 2021;13:1336–1366.
[33] Ludvigsson JF, Bergquist A, Montgomery SM, et al. Risk of diabetes and cardiovascular disease in patients with primary sclerosing cholangitis. J Hepatol 2014;60:802–808.
[34] Matsumoto N, Ogawa M, Matsuoka S, et al. Prevalence and risk factors of diabetes mellitus in patients with autoimmune hepatitis. Intern Med 2016;55:879–885.
[35] Lin YL, Yao T, Wang YW, et al. Association between primary biliary cholangitis with diabetes and cardiovascular diseases: a bidirectional multivariable Mendelian randomization study. Clin Res Hepatol Gastroenterol 2024;48:102419.
[36] **Lv D**, **Wang H**, Leng Y, et al. Association between diabetes mellitus and primary biliary cholangitis: a two-sample Mendelian randomization study. Front Endocrinol (Lausanne) 2024;15:1362584.
[37] Jensen MD, Jepsen P, Vilstrup H, et al. Increased cancer risk in autoimmune hepatitis: a Danish nationwide cohort study. Am J Gastroenterol 2022;117:129–137.
[38] **Braga MH**, **Cancado GGL**, Bittencourt PL, et al. Risk factors for cancer in patients with primary biliary cholangitis and autoimmune hepatitis and primary biliary cholangitis overlap syndrome. Ann Hepatol 2023;28:101105.
[39] Sharma R, Verna EC, Simon TG, et al. Cancer risk in patients with autoimmune hepatitis: a nationwide population-based cohort study with histopathology. Am J Epidemiol 2022;191:298–319.
[40] Lundberg Bave A, Bergquist A, Bottai M, et al. Increased risk of cancer in patients with primary sclerosing cholangitis. Hepatol Int 2021;15:1174–1182.
[41] Boonstra K, Bokelaar R, Stadhouders PH, et al. Increased cancer risk in a large population-based cohort of patients with primary biliary cirrhosis: follow-up for up to 36 years. Hepatol Int 2014;8:266–274.
[42] De Martin E, Michot JM, Papouin B, et al. Characterization of liver injury induced by cancer immunotherapy using immune checkpoint inhibitors. J Hepatol 2018;68:1181–1190.
[43] Coukos A, Vionnet J, Obeid M, et al. Systematic comparison with autoimmune liver disease identifies specific histological features of immune checkpoint inhibitor-related adverse events. J Immunother Cancer 2022;10.
[44] Lu M, Li J, Haller IV, et al. Factors associated with prevalence and treatment of primary biliary cholangitis in United States health systems. Clin Gastroenterol Hepatol 2018;16:1333–1341 e1336.
[45] Shamaa O, Ahmed A, Rupp L, et al. Beyond the surface: unveiling hidden hurdles to primary biliary cholangitis care. Cureus 2024;16:e64753.
[46] Sivakumar M, Gandhi A, Shakweh E, et al. Widespread gaps in the quality of care for primary biliary cholangitis in UK. Frontline Gastroenterol 2022;13:32–38.
[47] Torisu Y, Nakano M, Takano K, et al. Clinical usefulness of ursodeoxycholic acid for Japanese patients with autoimmune hepatitis. World J Hepatol 2017;9:57–63.
[48] Czaja AJ, Carpenter HA, Lindor KD. Ursodeoxycholic acid as adjunctive therapy for problematic type 1 autoimmune hepatitis: a randomized placebo-controlled treatment trial. Hepatology 1999;30:1381–1386.
[49] Schregel I, Papp M, Sipeki N, et al. Unmet needs in autoimmune hepatitis: results of the prospective multicentre European Reference Network Registry (R-LIVER). Liver Int 2024;44:2687–2699.
[50] Plagiannakos CG, Hirschfield GM, Lytvyak E, et al. Treatment response and clinical event-free survival in autoimmune hepatitis: a Canadian multicentre cohort study. J Hepatol 2024;81:227–237.
[51] Corpechot C, Carrat F, Gaouar F, et al. Liver stiffness measurement by vibration-controlled transient elastography improves outcome prediction in primary biliary cholangitis. J Hepatol 2022;77:1545–1553.
[52] Barner-Rasmussen N, Pukkala E, Jussila A, et al. Epidemiology, risk of malignancy and patient survival in primary sclerosing cholangitis: a population-based study in Finland. Scand J Gastroenterol 2020;55:74–81.

Keywords: Rare disease; Autoimmunity; Epidemiology; Prognosis; France.
Received 20 April 2025; received in revised form 11 July 2025; accepted 4 August 2025; Available online 11 August 2025

Journal of Hepatology, Volume *7*

Supplemental information

Epidemiology, comorbidities, treatments and outcomes of autoimmune liver diseases: A French nationwide study

Christophe Corpechot, Pierre Hornus, Mallory Cals, Pierre Rinder, Théo Marcille, Amina Malek, Karima Ben Belkacem, Farid Gaouar, Yasmina Chabane, Pierre-Antoine Corret, Paola Squarzoni, Pierre-Antoine Soret, Sara Lemoinne, Olivier Chazouillères, and Angela Leburgue

Epidemiology, comorbidities, treatments and outcomes of autoimmune liver diseases: A French nationwide study

Christophe Corpechot, Pierre Hornus, Mallory Cals, Pierre Rinder, Théo Marcille, Amina Malek, Karima Ben Belkacem, Farid Gaouar, Yasmina Chabane, Pierre Corret, Paola Squarzoni, Pierre Antoine Soret, Sara Lemoinne, Olivier Chazouillères, Angela Leburgue

Table of contents

Fig. S1 2

Fig. S2 3

Fig. S3. 4

Fig. S4. 5

Fig. S5. 6

Fig. S6 7

Fig. S7 8

Table S1. 9

Table S2 10

Table S3. 11

Table S4 12

Table S5 13

List of codes used for analysis. 14

Fig. S1. Flow chart of the study.

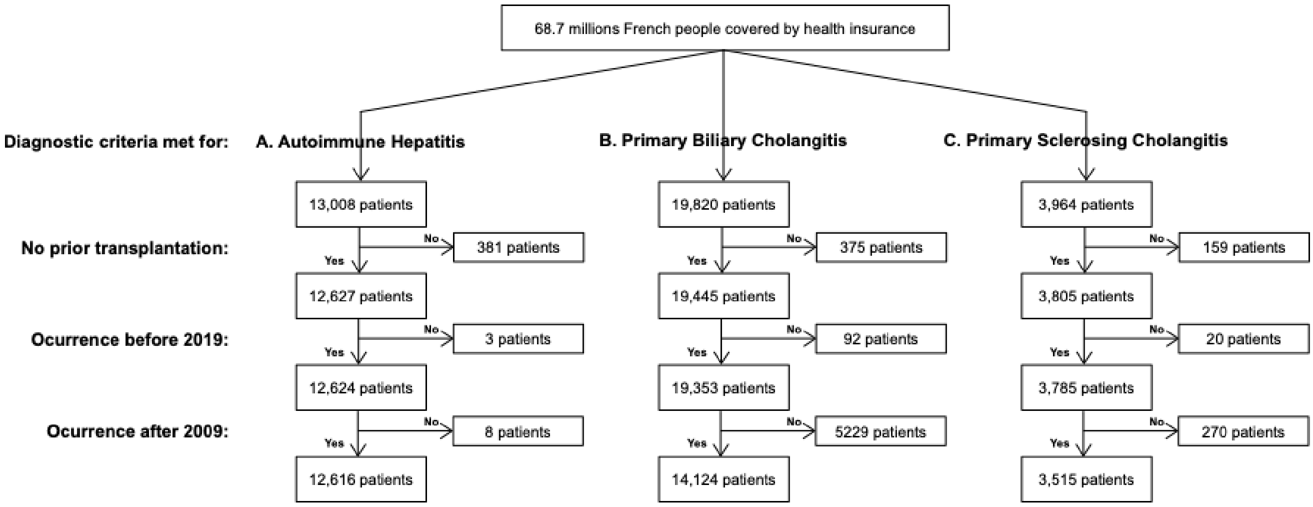

Fig. S2. Moran scatterplots of French departments for the prevalence of the three AILDs.

A. Autoimmune Hepatitis:

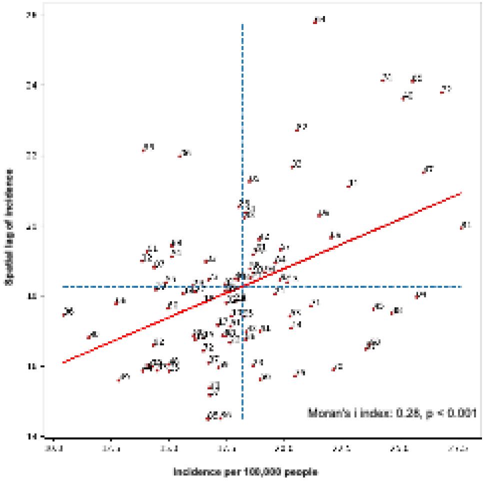

B. Primary Biliary Cholangitis:

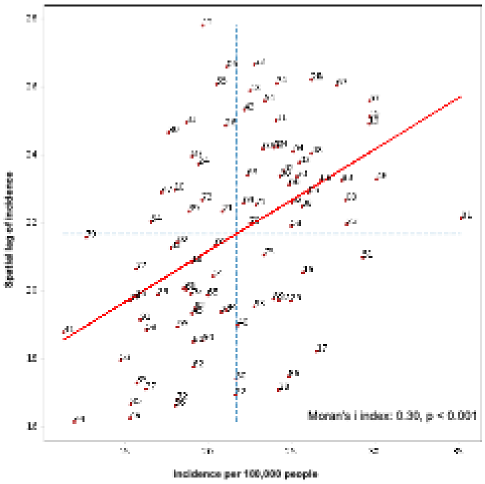

C. Primary Sclerosing Cholangitis:

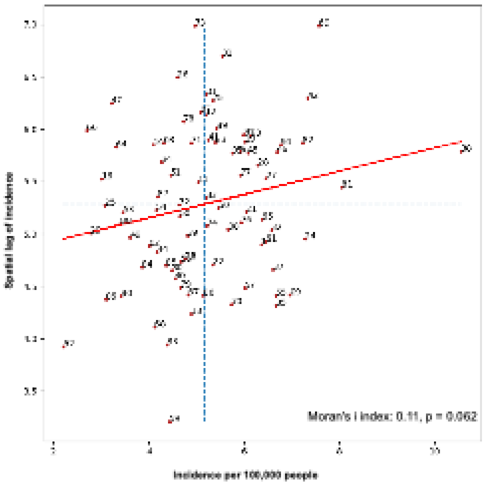

Fig. S3. Correlation between departmental prevalence and local density of hepato-gastroenterologists.

A. Autoimmune Hepatitis:

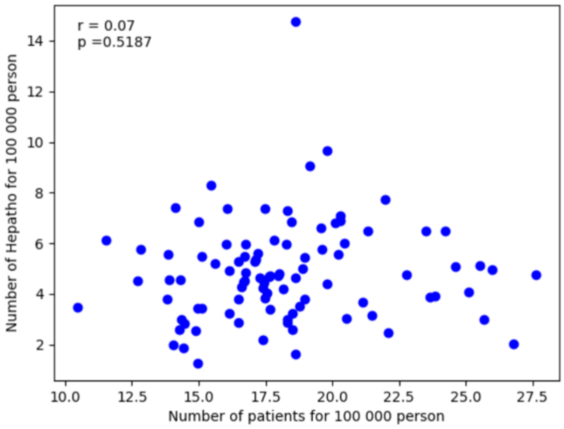

B. Primary Biliary Cholangitis:

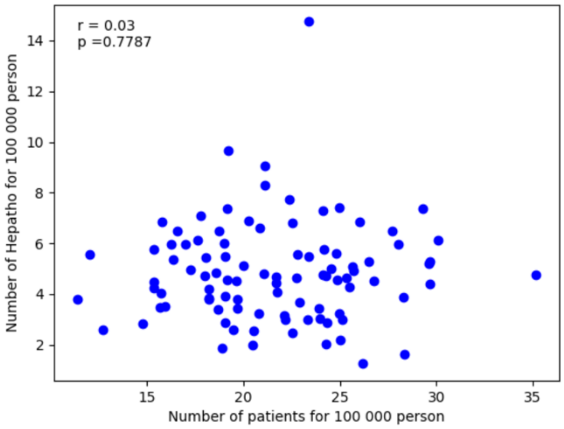

C. Primary Sclerosing Cholangitis:

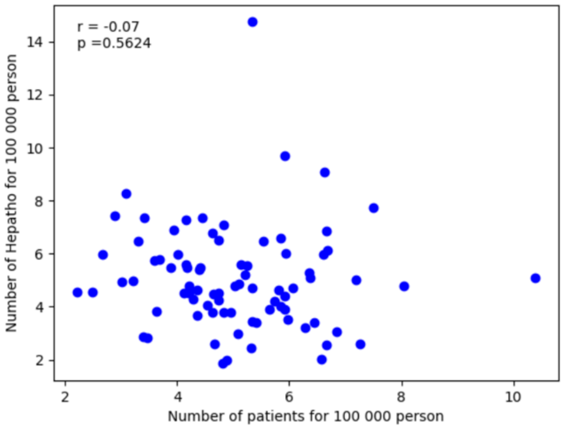

Fig. S4. Distribution of the number of consultations in the year following inclusion.

A. Autoimmune Hepatitis:

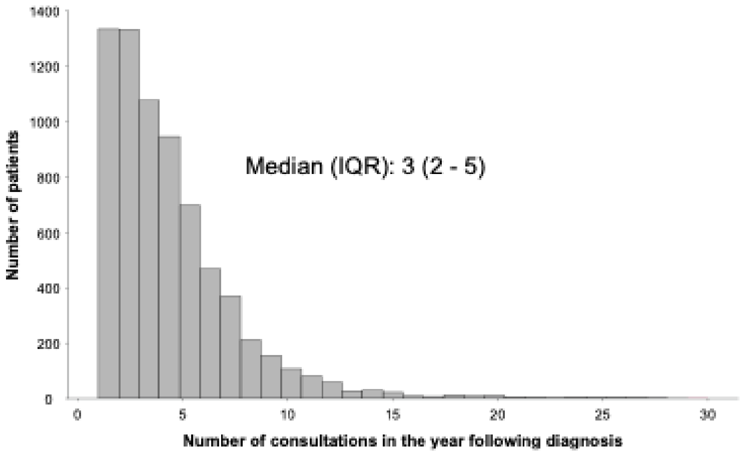

B. Primary Biliary Cholangitis:

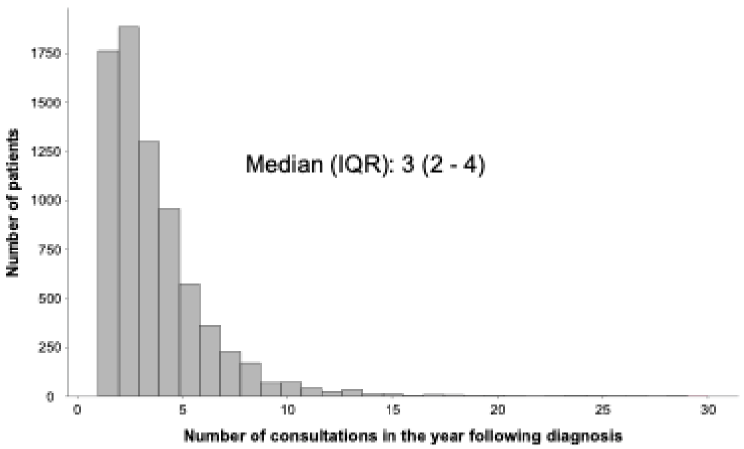

C. Primary Sclerosing Cholangitis:

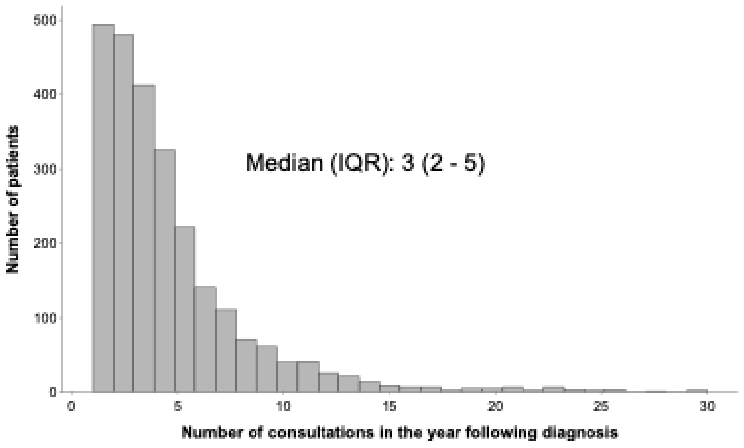

Fig. S5. Distribution of the number of hospital admissions in the year following inclusion.

A. Autoimmune Hepatitis:

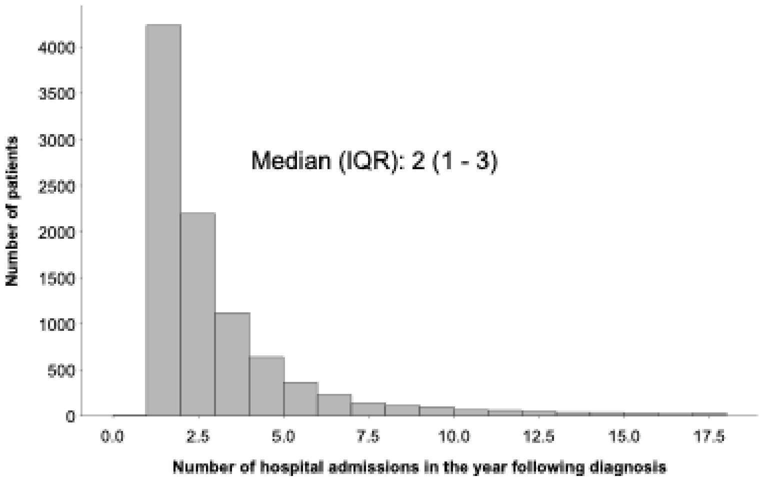

B. Primary Biliary Cholangitis:

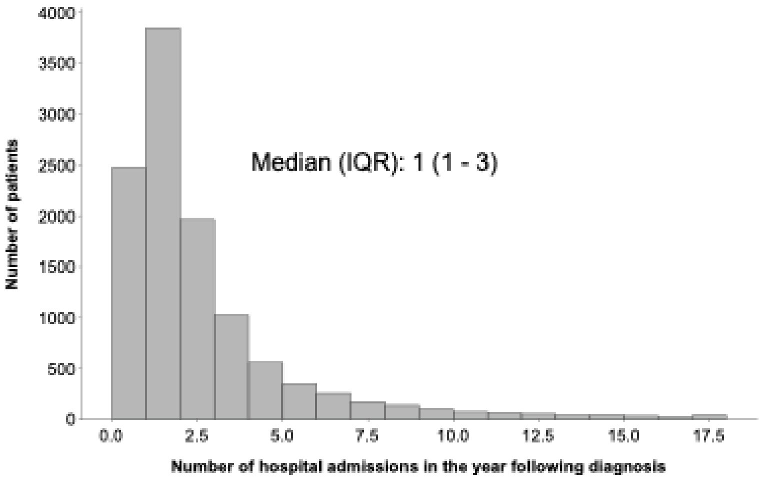

C. Primary Sclerosing Cholangitis:

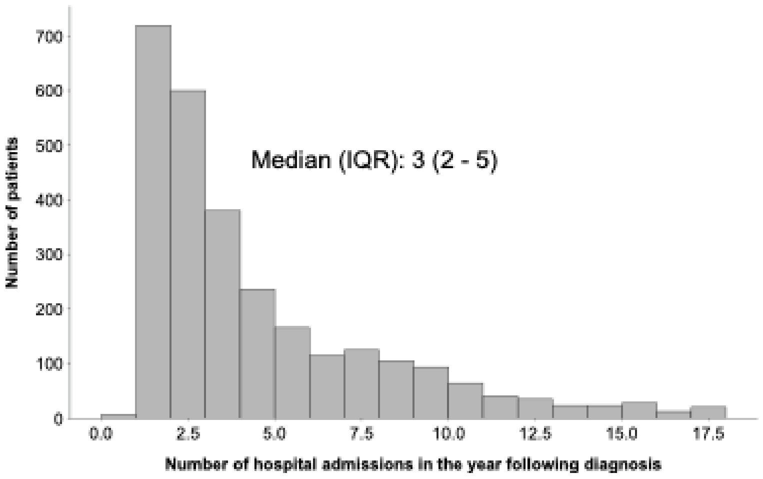

Fig. S6. Distribution of the number of inpatient days in the year following inclusion.

A. Autoimmune Hepatitis:

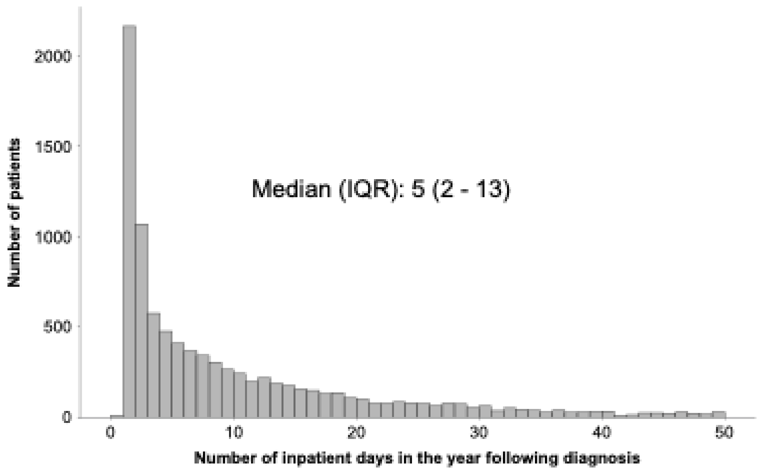

B. Primary Biliary Cholangitis:

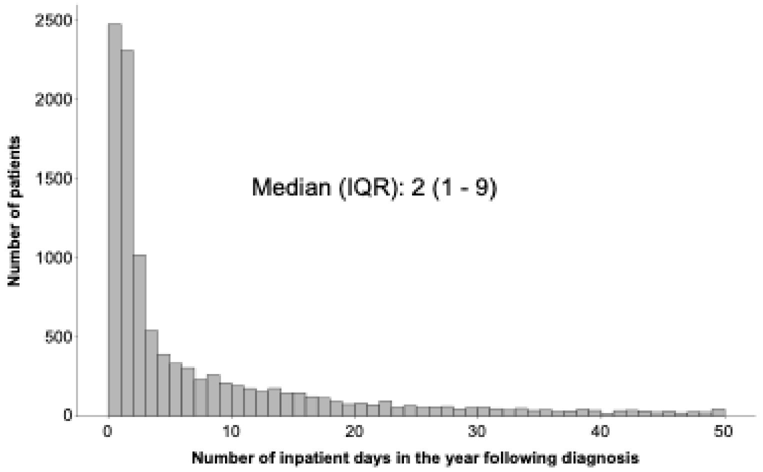

C. Primary Sclerosing Cholangitis:

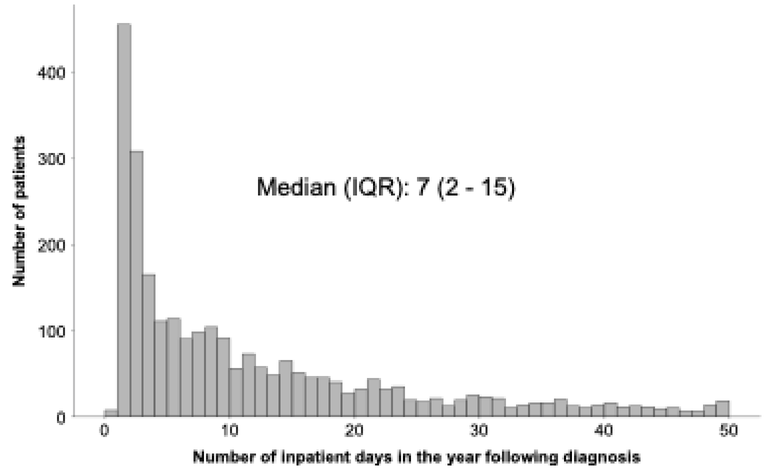

Fig. S7. Alluvial diagram of drugs dispensed in the year following inclusion.

A. Autoimmune Hepatitis:

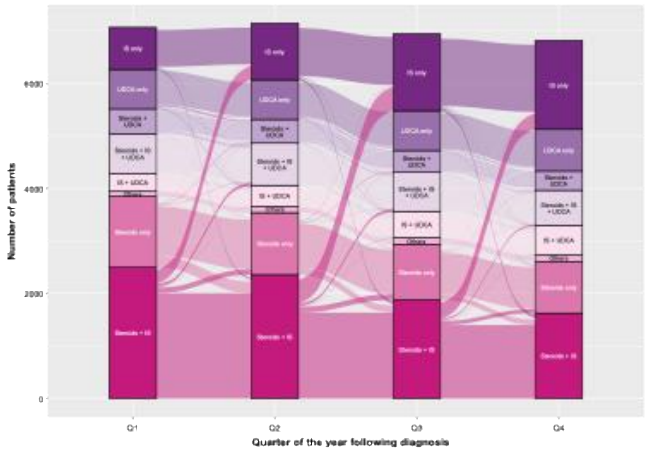

B. Primary Biliary Cholangitis:

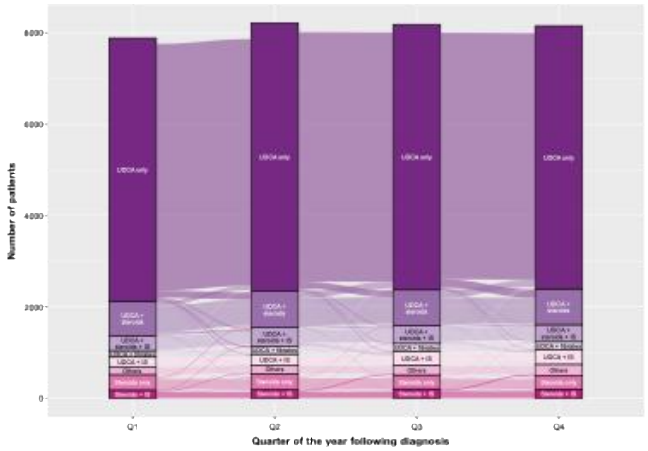

C. Primary Sclerosing Cholangitis:

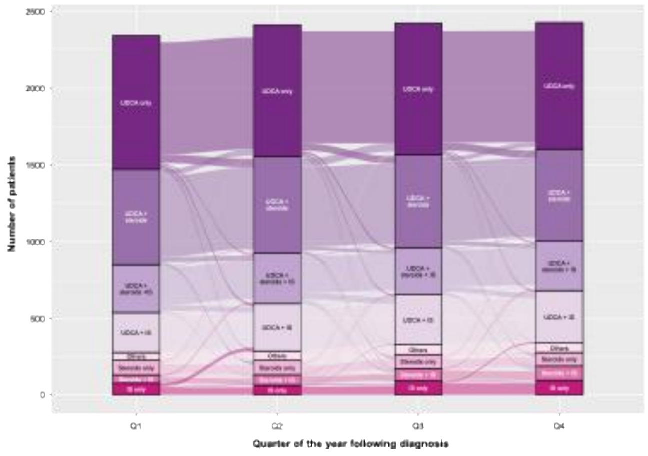

Table S1. Overall and disease-specific prevalence (percentage) of extrahepatic autoimmune conditions at the time of AILD diagnosis.

Condition	Overall	AIH	PBC	PSC
Autoimmune thyroiditis	2.93	3.50	2.70	1.80
Type 1 diabetes	2.52	2.29	2.83	2.09
Sjögren's syndrome	1.21	1.39	1.20	0.55
Systemic scleroderma	1.16	0.69	1.75	0.46
Rheumatoid arthritis	1.04	1.20	0.97	0.78
Systemic lupus erythematosus	0.73	1.20	0.42	0.32
Raynaud's syndrome	0.69	0.55	0.91	0.29
Ankylosing spondylitis	0.47	0.62	0.27	0.73
Psoriasis	0.44	0.55	0.35	0.41
Multiple sclerosis	0.34	0.48	0.24	0.20
Imm. thrombocytopenic purpura	0.26	0.32	0.22	0.17
Pernicious anemia	0.22	0.23	0.23	0.12
Celiac disease	0.21	0.29	0.13	0.23
Dermatomyositis / Polymyositis	0.18	0.25	0.14	0.06

Autoimmune diseases are ranked in descending order of overall prevalence. Those with an overall prevalence of less than 0.1% were excluded.

Table S2. The 10 most frequently reported active cancer codes in the year prior to inclusion.

	Designation	Occurrences
**AIH**		
C780	Secondary malignant lung tumor	76
C787	Secondary malignant tumor of the liver and intrahepatic bile ducts	70
C349	Malignant tumor of bronchus or lung, unspecified	62
C795	Secondary malignant tumor of bone and bone marrow	52
C439	Melanoma of the skin	45
C792	Secondary malignant skin tumor	44
C509	Malignant breast tumor, unspecified	43
C793	Secondary malignant tumor of the brain and cerebral meninges	40
C435	Malignant melanoma of the trunk	38
C786	Secondary malignant tumor of the retroperitoneum and peritoneum	38
**PBC**		
C509	Malignant breast tumor, unspecified	48
C787	Secondary malignant tumor of the liver and intrahepatic bile ducts	37
C61	Malignant prostate tumor	29
C443	Malignant tumor of the skin of the face, other and unspecified parts	23
C349	Malignant tumor of bronchus or lung, unspecified	22
C187	Malignant tumor of the sigmoid colon	22
C780	Secondary malignant lung tumor	21
C795	Secondary malignant tumor of bone and bone marrow	21
C50	Malignant breast tumor	21
C786	Secondary malignant tumor of the retroperitoneum and peritoneum	20
**PSC**		
C189	Malignant tumor of the colon, unspecified	13
C249	Malignant tumor of the bile ducts, unspecified	11
C787	Secondary malignant tumor of the liver and intrahepatic bile ducts	9
C20	Malignant tumor of the rectum	9
C187	Malignant tumor of the sigmoid colon	7
C779	Secondary, unspecified malignant tumor of a lymph node	7
C180	Malignant tumor of the cecum	6
C509	Malignant breast tumor, unspecified	6
C786	Secondary malignant tumor of the retroperitoneum and peritoneum	6

Table S3. Number of medicines dispensed and users in the year following inclusion (patients aged over 18 years with one year of data available after inclusion).

	No. of deliveries	No. of users	Percent. of users
**AIH (n=9,599)**			
Ursodeoxycholic acid	26,063	2,996	31.2%
Obeticholic acid	78	11	0.1%
Bezafibrate/fenofibrate	1,028	186	1.9%
Corticosteroids	47,399	5,729	59.7%
Prednisone	29,972	3,533	36.8%
Prednisolone	13,119	2,729	28.4%
Budesonide	7,910	1,565	16.3%
Immunosuppressives	43,820	5,853	59.9%
Azathioprine	31,571	4,940	51.5%
Mycophenolate mofetil	5,446	743	7.7%
Tacrolimus	2,312	218	2.3%
Ciclosporin	1,497	149	1.6%
**PBC (n=10,905)**			
Ursodeoxycholic acid	79,809	7,938	72.8%
Obeticholic acid	132	31	0.3%
Bezafibrate/fenofibrate	2,085	359	3.3%
Corticosteroids	17,607	3,680	33.8%
Prednisone	10,555	1,450	13.3%
Prednisolone	4,648	1,987	18.2%
Budesonide	1,791	363	3.3%
Immunosuppressives	10,971	1,319	11.7%
Azathioprine	4,357	725	6.7%
Mycophenolate mofetil	1,675	240	2.2%
Tacrolimus	1,277	110	1.0%
Ciclosporin	525	51	0.5%
**PSC (n=2,844)**			
Ursodeoxycholic acid	22,457	2,534	89.1%
Obeticholic acid	61	≤ 10	NA
Bezafibrate/fenofibrate	530	88	3.1%
Corticosteroids	6,624	1,239	43.6%
Prednisone	3,520	573	20.2%
Prednisolone	2,305	695	24.4%
Budesonide	947	203	7.1%
Immunosuppressives	9,787	1,184	35.7%
Azathioprine	3,782	577	20.3%
Mycophenolate mofetil	939	150	5.3%
Tacrolimus	1,055	112	3.9%
Ciclosporin	391	35	1.2%

Table S4. Survival rates and transplant-free survival rates after excluding patients whose follow-up since diagnosis was ≤ 1 year (landmark analysis).

	At 5 years	At 10 years
**AIH**		
Survival rate	89% (89% – 90%)	77% (75% – 79%)
Transplant-free survival rate	88% (87% – 89%)	75% (73% – 76%)
**PBC**		
Survival rate	84% (83% – 85%)	70% (68% – 72%)
Transplant-free survival rate	82% (82% – 83%)	68% (66% – 69%)
**PSC**		
Survival rate	90% (88% – 91%)	78% (75% – 81%)
Transplant-free survival rate	82% (81% – 84%)	66% (63% – 69%)

Table S5. The 10 most frequently reported diagnoses in death certificates (patients whose death certificate included at least one ICD10 code for liver disease)

	ICD-10 code	No. of reports	%. of deaths
**AIH (n=593)**			
Autoimmune hepatitis	K754	377	30.3%
Respiratory arrest	R092	206	16.6%
Hepatic failure	K729	170	13.7%
Unspecified cirrhosis	K746	170	13.7%
Other general symptoms and signs	R688	154	12.4%
Severe sepsis with septic shock	R652	110	8.9%
Sepsis, unspecified organism	A419	109	8.8%
Secondary malignant neoplasm	C799	77	6.2%
Heart failure, unspecified	I509	76	6.1%
Pneumonia, unspecified organism	J189	76	6.1%
**PBC (n=1,061)**			
Respiratory arrest	R092	375	17.6%
Unspecified cirrhosis	K746	369	17.3%
Primary biliary cirrhosis	K743	324	15.2%
Hepatic failure	K729	291	13.7%
Liver cell carcinoma	C220	207	9.7%
Other general symptoms and signs	R688	186	8.7%
Sepsis, unspecified organism	A419	158	7.5%
Ascites	R18	156	7.3%
Heart failure, unspecified	I509	151	7.0%
Severe sepsis with septic shock	R652	133	6.2%
**PSC (n=125)**			
Cholangitis	K830	63	22.9%
Intrahepatic bile duct carcinoma	C221	44	16.0%
Hepatic failure	K729	41	14.9%
Unspecified cirrhosis	K746	41	14.9%
Respiratory arrest	R092	39	14.2%
Other general symptoms and signs	R688	34	12.4%
Sepsis, unspecified organism	A419	30	10.9%
Secondary malignant neoplasm	C799	27	9.8%
Ulcerative colitis, unspecified	K519	27	9.8%
Severe sepsis with septic shock	R652	24	8.7%

List of codes used for analysis.

Codes used to include patients in the PSC cohort

ICD-10 code	Diagnosis
K50	Crohn's disease
K51	Ulcerative colitis

CCAM code	Imaging
ZCQN002	Magnetic resonance imaging of the abdomen or pelvis, without intravenous injection of contrast medium
ZCQJ004	Magnetic resonance imaging of the abdomen or pelvis, with intravenous injection of contrast medium
ZCQJ005	Magnetic resonance imaging of the abdomen and pelvis, with intravenous injection of contrast medium
ZCQN001	Magnetic resonance imaging of the abdomen and pelvis, without intravenous injection of contrast medium
ZCQH001	Computed tomography scan of the abdomen and pelvis, with intravenous injection of contrast medium
ZCQH002	Computed tomography scan of the abdomen or pelvis, with intravenous injection of contrast medium
ZCQK004	Computed tomography scan of the abdomen and pelvis, without intravenous injection of contrast medium
ZCQK005	Computed tomography scan of the abdomen or pelvis, without intravenous injection of contrast medium
ZCQM008	Transcutaneous ultrasound of the abdomen
ZCQM001	Transcutaneous ultrasound of the abdomen, with Doppler ultrasound of the digestive vessels
ZCQM005	Transcutaneous ultrasound of the abdomen, with transcutaneous ultrasound of the pelvis
ZCQM004	Transcutaneous ultrasound of the upper abdomen with ultrasound of the digestive vessels
ZCQM002	Transcutaneous ultrasound of the abdomen, with transcutaneous ultrasound of the pelvis and Doppler ultrasound of the digestive vessels

Codes used for cancer detection:

ICD-10 codes	Diagnosis
C220	Liver cell carcinoma
C221	Intrahepatic bile duct carcinoma

C227	Other specified carcinomas of liver
C229	Malignant neoplasm of liver, not specified as primary or secondary
C240	Malignant neoplasm of extrahepatic bile duct
C18	Malignant neoplasm of colon
C**	Malignant neoplasm
D0*	Carcinoma in situ

Codes used to analyze drug use:

**ATC code**	**Label**
A05AA02	Ursodeoxycholic acid (UDCA)
A02AA04	Obeticholic acid
C10AB	Bezafibrate / fenofibrate
H02	Corticosteroids
H02AB06	Prednisolone
H02AB07	Prednisone
A07EA06	Budesonide
L04	Immunosuppressive drug
L04AA06	Mycophenolate Mofetil
L04AX01	Azathioprine
L04AD02	Tacrolimus
L04AD01	Cyclosporine

**ATC code**	**Label**
A07EC02	Mesalazine
L04AB01	Etanercept
L04AB02	Infliximab
L04AB04	Adalimumab
L04AB05	Certolizumab
L04AB06	Golimumab
L04AA33	Vedolizumab
J04AB02	Rifampicin

Codes used for the number of abdominal and liver scans performed during the first year after detection of the disease and for the six-monthly detection of cirrhosis:

**CCAM code**	**Label**

ZCQM008	Transcutaneous ultrasound of the abdomen
ZCQH001	CT scan of the abdomen and pelvis, with intravenous injection of contrast medium
ZCQM002	Transcutaneous ultrasound of the abdomen, with transcutaneous ultrasound of the pelvis and Doppler ultrasound of the digestive vessels
ZCQJ004	Magnetic resonance imaging of the abdomen or pelvis, with intravenous injection of contrast medium
ZCQM010	Transcutaneous ultrasound of the upper abdomen and pelvis
ZCQM004	Transcutaneous ultrasound of the upper abdomen with Doppler ultrasound of the digestive vessels
ZCQM006	Transcutaneous ultrasound of the upper abdomen
ZCQM001	Transcutaneous ultrasound of the abdomen, with Doppler ultrasound of the digestive vessels
ZCQM011	Transcutaneous ultrasound of the upper abdomen and pelvis and Doppler ultrasound of digestive vessels
ZCQN001	Magnetic resonance imaging of the abdomen and pelvis, without intravenous injection of contrast medium
ZCQJ005	Magnetic resonance imaging of the abdomen and pelvis, with intravenous injection of contrast medium
ZCQN002	Magnetic resonance imaging of the abdomen or pelvis, without intravenous injection of contrast medium
ZCQK004	CT scan of the abdomen and pelvis, without intravenous injection of contrast medium
ZCQK005	CT scan of the abdomen or pelvis, without intravenous injection of contrast medium
ZCQM005	Transcutaneous ultrasound of the abdomen, with transcutaneous ultrasound of the pelvis
ELQJ003	Magnetic resonance imaging of vessels in the abdomen and/or pelvis, with magnetic resonance imaging of vessels in the limbs
ELQJ001	Magnetic resonance imaging of vessels in the abdomen and/or pelvis
ELQH002	CT scan of vessels in the abdomen and/or pelvis
ZCQH002	CT scan of the abdomen or pelvis, with intravenous injection of contrast medium
ECQH011	CT scan of vessels in the thorax and/or heart, with CT scan of vessels in the abdomen and/or pelvis

ZCQM005	Transcutaneous ultrasound of the abdomen, with transcutaneous ultrasound of the pelvis
ELQH001	Scan of liver vessels to study vascularisation at at least 3 different times
HLQM001	Transcutaneous ultrasound of the liver and bile ducts
HLQM002	Liver stiffness measurement using ultrasound pulse elastograph

Codes used for the number of endoscopies and colonoscopies performed during the first year after detection of the disease:

**CCAM code**	**Label**
HHQE	Colonoscopy
HEQE002	Oeso-gastro-duodenal endoscopy
HMQA001	Intraoperative endoscopy of the bile ducts, by transcystic approach or choledochotomy
HMQH003	Retrograde cholangiography with infundibulotomy or pre-cutting of the major duodenal papilla, by oeso-gastro-duodenoscopy
HMQH007	Retrograde cholangiography, by oeso-gastro-duodenoscopy
HMQH004	Cholangiography, using transcutaneous injection of a contrast agent into the bile ducts, with ultrasound and/or radiological guidance.
HMQH006	Cholangiography, by injection of contrast product into an external biliary drain

Codes used for different types of hospitalization and complications of cirrhosis:

**CCAM & ICD10 code**	**Procedure or diagnosis**
HLN HLC HLJ HLF HLS HLH	Hepato-biliary surgery
NBR_SUP_REA	Stays in intensive care
NBR_SUP_STF	Stays in intensive care unit
YYYY020 YYYY015	Stays in intensive care (procedure)
Z511	Chemotherapy session
HLNC003 HLNM001 HLNK001 HLNA007 HLNN900	Hepatic tumour destruction by radiofrequency
Z944	Liver transplantation (diagnosis)

HLEA001 HLEA002 HGEA002 HGEA004	Liver transplantation (procedure)
HMQH HNQH001 HNQH003	Retrograde cholangio-pancreatography
EHNE002 EHNE001	Endoscopic ligation of oesophageal varices
EHCF002	Transjugular intrahepatic portosystemic shunt (TIPS)
EDLF017 EDLF016 EDLL001	Liver embolisation
R18	Ascites
K922	Digestive bleeding
G934	Hepatic encephalopathy
K767	Hepato-renal syndrome
J948	Hydrothorax
I272	Porto-pulmonary hypertension
C220 C221 C227 C229 C240	Primary liver cancer

Codes used for different types of work stoppage:
The variable used to calculate the number of days off work is PRS_ACT_NBR.

**Code**	**Meaning**
6110	Normal daily allowances plus 6 months
6111	Normal daily allowances minus 3 months
6112	Normal daily allowances plus 3 months
6113	Reduced daily allowances minus 3 months
6114	Reduced daily allowances plus 3 months
6115	Increased daily allowances minus 3 months
6116	Increased daily allowances plus 3 months
6117	Partial daily allowances, loss of pay minus 3 months
6118	Partial daily allowances, loss of pay plus 3 months
6119	Increased daily allowances plus 6 months
